# Control of Photoconversion Yield in Unidirectional Photomolecular Motors by Push–Pull Substituents

**DOI:** 10.1021/jacs.3c06070

**Published:** 2023-08-30

**Authors:** Palas Roy, Andy S. Sardjan, Wojciech Danowski, Wesley R. Browne, Ben L. Feringa, Stephen R. Meech

**Affiliations:** † School of Chemistry, University of East Anglia, Norwich NR4 7TJ, U.K.; ‡ Centre for Systems Chemistry, Stratingh Institute for Chemistry, University of Groningen, 9747 AG Groningen, The Netherlands; § Molecular Inorganic Chemistry, Stratingh Institute for Chemistry, University of Groningen, 9747 AG Groningen, The Netherlands; ∥ School of Basic Sciences, Indian Institute of Technology Bhubaneswar, Bhubaneswar, Odisha 752050, India; ⊥ University of Strasbourg, CNRS, ISIS UMR 7006, 8 allée Gaspard Monge, F-67000 Strasbourg, France

## Abstract

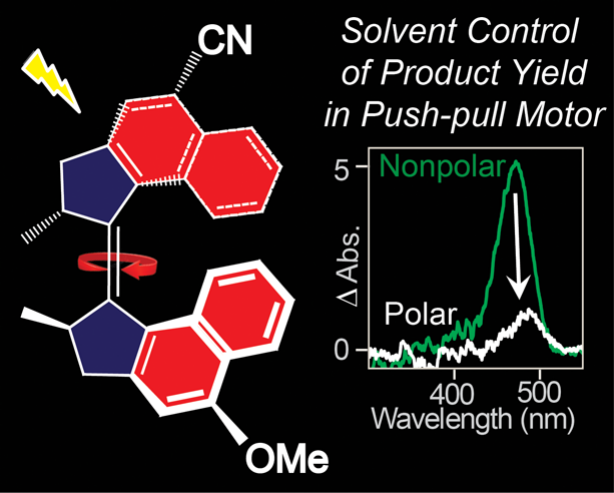

Molecular motors based on the overcrowded alkene motif convert light energy into unidirectional mechanical motion through an excited state isomerization reaction. The realization of experimental control over conversion efficiency in these molecular motors is an important goal. Here, we combine the synthesis of a novel “push–pull” overcrowded alkene motor with photophysical characterization by steady state and ultrafast time-resolved electronic spectroscopy. We show that tuning of the charge transfer character in the excited state has a dramatic effect on the photoisomerization yield, enhancing it to near unity in nonpolar solvents while largely suppressing it in polar solvents. This behavior is explained through reference to solvent- and substituent-dependent potential energy surfaces and their effect on conical intersections to the ground state. These observations offer new routes to the fine control of motor efficiency and introduce additional degrees of freedom in the synthesis and exploitation of light-driven molecular motors.

## Introduction

**Figure 1:**
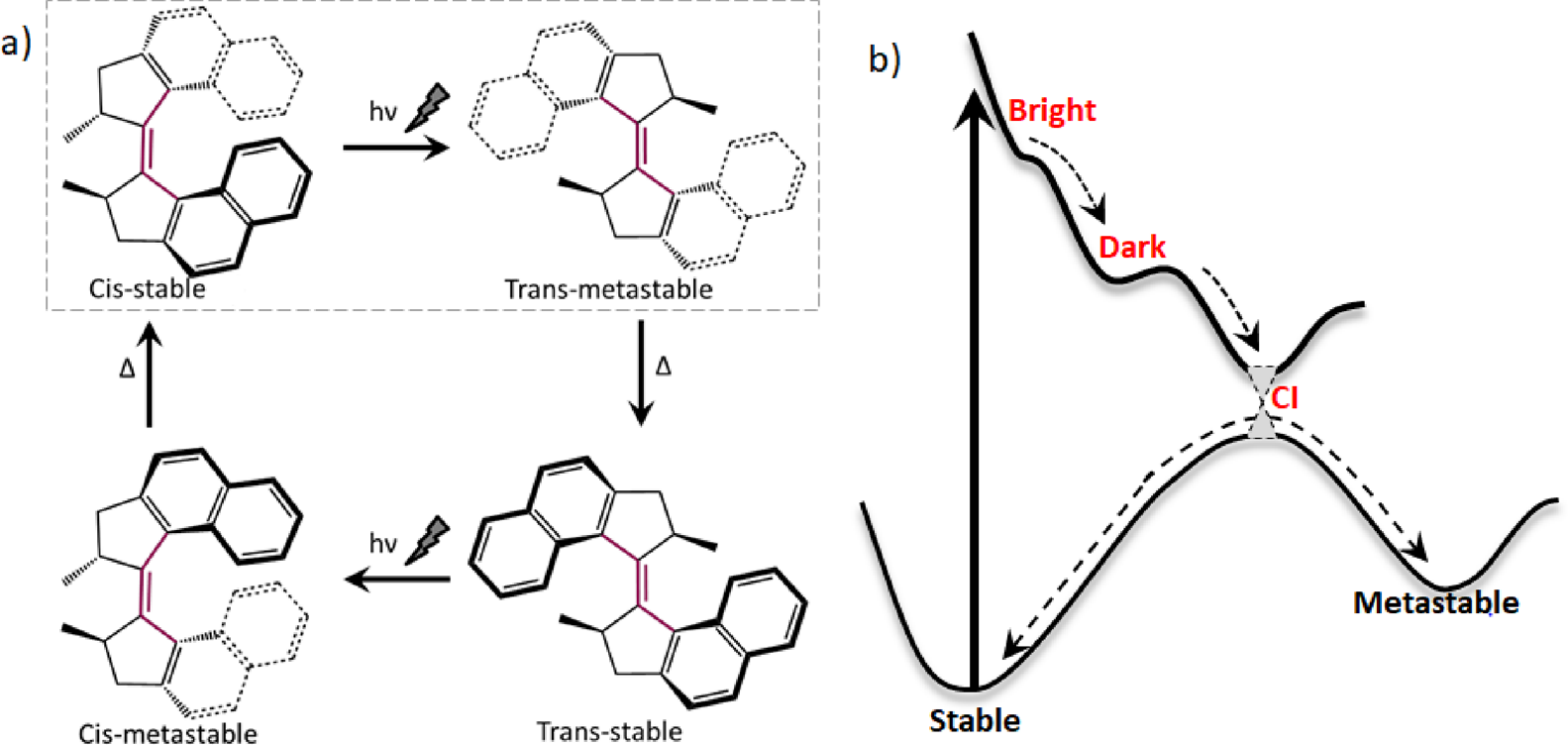
(a) Photocycle of a first-generation motor. *h*ν indicates a light-driven excited state isomerization step, while Δ indicates a thermal helix inversion, which proceeds over the lower barrier to generate the new isomer. The present study focuses on the cis-stable to trans-metastable photoreaction (boxed). (b) A generic potential energy diagram for light-driven motor function is shown in which initial photoexcitation leads to a Franck–Condon bright state, which then forms a picosecond lifetime dark state that decays through a conical intersection where population bifurcates to either the initial reactant or the metastable product.

Unidirectional rotation driven by light absorption was first demonstrated in C2-symmetric overcrowded alkenes possessing two stereocenters more than 20 years ago.^1,2^ Through a combination of absorption spectroscopy and circular dichroism, the mechanism was revealed to be a four-state cycle in which excited state isomerization to yield a metastable ground state product was followed by a helix inversion to preferentially form a new stable isomer. This isomer can then undergo a further photoisomerization/inversion reaction to yield one full rotation;^1,2^ the photocycle of the prototypical first-generation motor (1GM) is shown in Figure 1a.

Efforts to increase the rate of the rate-limiting helix inversion step through modifications to the chemical structure resulted in a second generation of molecular motors with a single stereocenter, which were shown to support MHz rotation rates.^3−6^ Recently, third-generation motors were developed, comprising two coupled second-generation motors with a single pseudo stereocenter, which are capable of supporting light-driven translational motion on a surface.^7−9^ The efficiency of all these molecular motors is a product of the helix inversion rate and the quantum yield of isomerization.^10,11^ While control of the former has been successfully demonstrated by synthetic variation,^4^ manipulation of the latter requires knowledge of excited state reaction pathways, which have proven less amenable to control.

There have been numerous measurements of excited state dynamics in molecular motors, and the picture that emerges is common to all three generations (Figure 1b).^12−20^ Electronic excitation localized on the sterically strained C=C “axle” populates a Franck–Condon state that undergoes a rapid (typically <200 fs) relaxation on the excited state potential energy surface to populate an intermediate excited state with a greatly reduced transition moment, a “dark” state. This dark state undergoes further structural relaxation on a picosecond timescale and ultimately passes through a conical intersection (CI) to populate either the metastable state or the original ground state. It is this bifurcation that determines the quantum yield of photochemical isomerization.

Excited state dynamics of molecular motors have also been investigated through quantum chemical calculations.^10,21−26^ These suggest that key coordinates in the excited state relaxation are rotation about the ethylenic double bond and pyramidalization at the ethylenic carbon atoms. Ultrafast motion along these coordinates leads to the dark state. From the dark state minimum on the excited state surface, the motor accesses CIs with the electronic ground state via low energy barriers. It has been proposed that engineering the location and nature of CIs is critical in controlling the quantum yield of isomerization.^23^

Recently, it was shown that the first-generation motor (Figure 1a) has a dark state lifetime that is a strong function of solvent polarity.^12^ Building on earlier models of ethylene and stilbene isomerization,^27−29^ this was assigned to polar solvent stabilization of a charge transfer (CT) configuration arising from the “sudden polarization” that occurs during excited state isomerization of ethylenic double bonds. Polar solvents preferentially stabilize this polar state, leading to suppression of a barrier along the reaction coordinate, and thus, the faster excited state decay is observed. Although the excited state lifetime of the first-generation motor was markedly decreased in polar solvents, this did not correspond to a significant change in the yield of the photoproduct, *i.e.*, solvent control of the isomerization quantum yield was not realized. However, inspired by this evidence of the importance of CT character in the reaction coordinate, we designed a first-generation motor with strategically located electron donating (methoxy) and withdrawing (cyano) groups in conjugation with the isomerizing “axle” double bond, a “push–pull” motor. This work builds on a recent study of a push–pull motor based on the second-generation core. In that case, it was shown that push–pull substituents greatly modified the energetics of the ground state helix inversion reaction and introduced a significant solvent polarity effect upon it.^30^ Here, we show that such synthetically engineered CT character in a first-generation motor core has a remarkable effect on the excited state dynamics, specifically the quantum yield of the cis-stable to trans-metastable isomerization reaction, rendering it much more sensitive to solvent polarity than for the unsubstituted motor. Because the fundamental picture of excited state dynamics (Figure 1b) is common to all motors, it is anticipated that this result will also be applicable to later generations of motors.

## Results and Discussion

**Figure 2:**
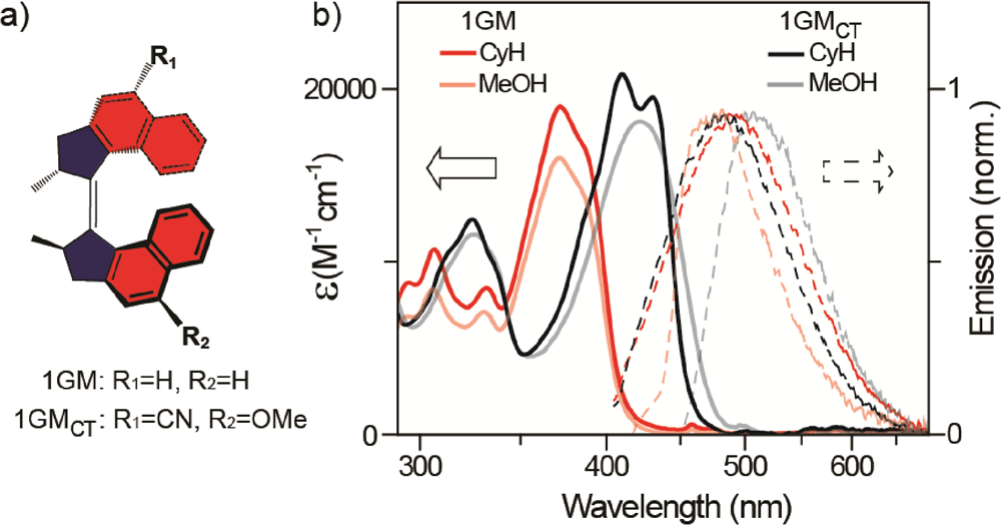
(a) Chemical structures of 1GM and 1GM_CT_. The ethylenic bond is in the plane of the paper, and lighter/darker bonds indicate orientation below/above the page. (b) Absorption (solid lines) and emission (dashed lines) as a function of solvent for 1GM (red) or 1GM_CT_ (black).

The structure of the push–pull motor is shown in Figure 2a, and its synthesis and characterization are described in the Experimental Methods sections and Supporting Information. We label this motor 1GM_CT_ to highlight its first-generation motor core (1GM) and CT substituents.

Figure 2b compares the absorption and emission spectra of 1GM_CT_ with 1GM in nonpolar cyclohexane and polar methanol. Emission spectra were recorded with a fast scan method (Supporting Information) to avoid contributions from the buildup of photoproduct isomers (Figure 1). The absorption spectrum of 1GM_CT_ in cyclohexane and methanol is markedly red-shifted from 1GM (by 37 and 50 nm, respectively). However, the maximum extinction coefficient is slightly increased in 1GM_CT_, suggesting that the electronic transition from the ground state retains a ππ* rather than CT character (which is typically associated with a reduced transition moment). There is also a loss of vibronic structure in methanol, suggesting additional interactions between the H-bonding solvent and 1GM_CT_ contributing to spectral broadening. Turning to the fluorescence, in agreement with earlier studies, the emission spectra of 1GM are broad featureless and only weakly dependent on solvent polarity, showing an 8 nm blue shift in emission between nonpolar cyclohexane and polar methanol. In contrast, the emission spectrum of 1GM_CT_ is a strong function of solvent polarity, exhibiting a marked red shift in the emission spectrum, from 482 nm in cyclohexane to 509 nm in methanol. This solvatochromism shows that the push–pull substituents have imparted a significant CT character to the emissive state. The fluorescence quantum yields were also determined and are presented in Figure S16 and Table S2. As expected, the yields are uniformly low with methanol being lower than cyclohexane and 1GM greater than 1GM_CT_.

The absolute quantum yields for photoconversion to the metastable trans isomers of 1GM and 1GM_CT_ from their stable cis isomers (Figure 1a) were measured by an initial rate method using in situ NMR photolysis and an *o*-nitrobenzaldehyde actinometer; the method, which assumes conversion from trans metastable to trans stable (Figure 1a), is described in detail in the Supporting Information. Yield data are presented for the two motors in a range of nonpolar and polar solvents in Table 1; some data for 1GM were presented earlier using different methods.^12^ There is generally good agreement that the photoconversion quantum yield for 1GM is high in nonpolar solvents, with reported values ranging from 0.85 to 0.59.^12,19^ The present data extend these observations to confirm that the photoconversion quantum yield of 1GM does not show a significant solvent polarity dependence (0.6 ± 0.1) in all of the solvents studied. The data for 1GM_CT_ are very different. In nonpolar cyclohexane, the photoconversion yield is enhanced to a value near unity, the highest value reported (or possible) for any photomolecular motor. In sharp contrast, in polar methanol, the photoconversion quantum yield is reduced more than 10-fold. Even in chloroform (which has a much lower dielectric constant than methanol), the yield is similarly greatly reduced. In addition to its moderate polarity, CHCl_3_ is also highly polarizable. To distinguish the effects of these solvent properties, the photoconversion yield was measured in the polarizable but weakly polar toluene. In this case, a high photoconversion yield (0.9) was observed. Thus, these data demonstrate that the isomerization yield of first-generation motors can be modified by CT substituents, introducing a remarkable solvent polarity dependence.

**Table 1:** Photochemical Isomerization Yields for Cis Stable to Trans Metastable Reaction of 1GM and 1GM_CT_ as a Function of the Solvent Dielectric Constant, ε^a^

	CyH (C_6_D_12_)	toluene (C_7_D_8_)	ethanol (C_2_D_5_OD)	methanol (CD_3_OD)	chloroform (CDCl_3_)
ε	2.0	2.4	24.5	32.7	4.7
1GM	0.64	0.54	0.69^c^	0.42^b^	0.68
1GM_CT_	0.99	0.90	n.d.	0.05	0.04

a The excitation wavelength was 365 nm.

b Accuracy of measurements in methanol was compromised by low solubility.

c The result for 1GM ethanol was obtained by a different method^12^ but is in good agreement with the present methanol result.

**Figure 3:**
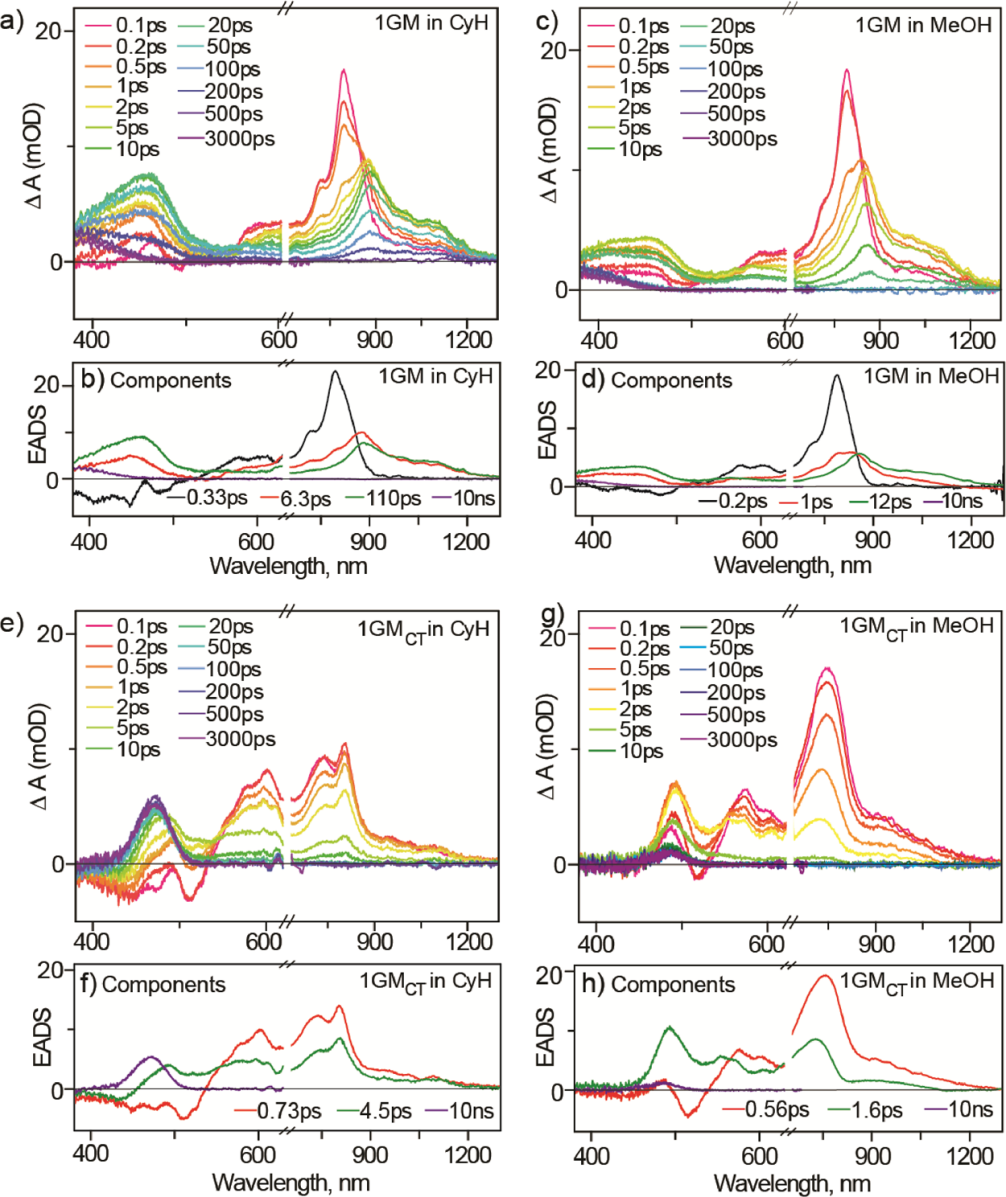
Time evolution of the transient absorption spectra of (a) 1GM in cyclohexane, (c) 1GM in methanol, (e) 1GM_CT_ in cyclohexane, and (g) 1GM_CT_ in methanol. The corresponding evolution-associated difference spectra (EADS) are shown in panels (b, d, f, h), respectively. The “10 ns” component represents a long-lived product state with an arbitrarily long lifetime beyond the maximum pump-probe delay time.

These stationary state data show that the introduction of electron donor–acceptor substituents dramatically alters the photoconversion yield in the motor photocycle. The origin of this behavior was investigated by transient absorption (TA) spectroscopy applied to 1GM and 1GM_CT_. TA spectra were measured in methanol and cyclohexane in a probe window of 380–1250 nm. The data for 1GM in cyclohexane and methanol are presented in Figure 3a and Figure 3c, respectively.

The evolution follows the mechanism set out in Figure 1b. The intense narrow excited state absorption (ESA) feature near 800 nm and the stimulated emission near 500 nm are associated with the Franck–Condon (FC) bright state, and both decay in a few hundred femtoseconds to form the dark state, which has a broader, red-shifted ESA rising at wavelengths above 900 nm, no stimulated emission, and an additional absorption near 450 nm. The dark state decay is non-single exponential with few ps to ca. 100 ps decay times. The decay is faster in the polar solvent, consistent with the emission quantum yields (Table S2) and lifetimes described in the Supporting Information and in earlier measurements,^12^ where a solvent-dependent barrier height was described. In both solvents, a product, the metastable trans ground state isomer, is formed from the dark state with an absorption near 400 nm (Figure 3a,c). This is most obvious in the global analysis of the TA data (Figure 3b,d). Here, the data are fit to a sequential model of evolution from the bright to dark state, which undergoes a two-step (non-single exponential) decay to the final state yielding the corresponding evolution-associated difference spectra (EADS). Two nanoseconds after excitation, the 400 nm absorbing final state is the only contribution to the transient absorption, so its amplitude is indicative of the metastable state photoproduct yield. This product yield for 1GM after 3 ns is approximately independent of solvent polarity, as expected from steady-state data, Table 1 (Figure 3b,d).

The TA data for 1GM_CT_ are strikingly different to those for 1GM. Figure 3e and Figure 3g show the data in cyclohexane and methanol, respectively, while the corresponding EADS are plotted in Figure 3f,h. The most obvious feature is the very high yield of the metastable state (ca. 430 nm) after 3 ns in cyclohexane and its very low yield in methanol, consistent with steady state data (Table 1). In addition, the ESA spectra are dependent on solvent polarity, showing a reduced structure in methanol. These results confirm that the effect of CT substituents on the product yield is through changes in the excited state potential energy surfaces. This is more evident when comparing the ultrafast dynamics of 1GM and 1GM_CT_. In the latter, there is no measurable rise time for a dark state. Instead, the kinetics exhibit only a non-single exponential monotonic decay such that one less component is required in the sequential global analysis to fit the data (compare EADS in Figure 3b,d with Figure 3f,g); the time constants from the global analysis are included in Figure 3. Thus, the dark state and the FC state are no longer kinetically distinct in 1GM_CT_. The absence of a clear FC to dark state evolution is confirmed by measurements of the wavelength-resolved ultrafast time-resolved fluorescence (Figure S14). In these, the 1GM_CT_ fluorescence decay is observed to be emission wavelength-independent, unlike for 1GM where the FC state contributes a rapid decay on the short wavelength side of the emission (refs (12,31) and Figure S14). Thus, in 1GM_CT_, the FC state has merged with the earliest appearance of the dark state (or has relaxed to it in <50 fs). However, the non-single exponential decay of the excited state in 1GM_CT_ persists and remains associated with a decrease in S_1_–S_0_ transition moment with time as the stimulated emission contributes only to the fastest component in the EADS (Figure 3e–h).

We have extended the TA measurements to derivatives of 1GM with one-push (OMe) or two-push and two-pull (CN) substituents. Qualitatively, these behave in the same way as 1GM (Figure S17). In particular, we have been able to determine the solvent-dependent yield from the relative amplitudes of the long-time TA in methanol and cyclohexane. For each derivative, the relative yield falls between 0.8 and 1.5 (Table S3). It is only for 1GM_CT_ that a large change (to 6) is observed; evidently, both a push and a pull substituent are required to achieve solvent control.

**Figure 4:**
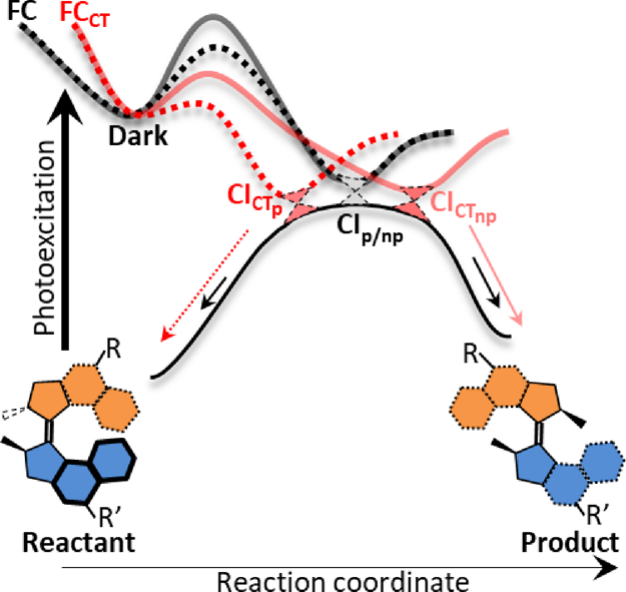
Schematic potential energy surfaces for the excited state reaction of 1GM (black) and 1GM_CT_ (red) in nonpolar (full line) and polar (dashed line) solvents. The reaction proceeds from the distinct Franck–Condon states to the dark state with no barrier. The dark state goes over a solvent- and substituent-dependent barrier to access a CI with the ground state. For 1GM, the CI is essentially independent of the solvent and slightly favors access to the product rather than the initial reactant isomer. In 1GM_CT_, the barrier is lower than for 1GM and more solvent-sensitive. This leads to a solvent-dependent CI with the ground state. In nonpolar (NP) solvents, the CI slope and location are such that the product is overwhelmingly favored. In polar (P) solvents, the CI favors the reactant channel.

Collectively, these data demonstrate that the push–pull substituents have introduced a new means of controlling the isomerization yield in molecular motors and that the control arises from changes in the excited state potential energy surface. The entire data set can be explained qualitatively by CT substituents introducing a solvent polarity-dependent asymmetry into the excited state dynamics. The effect is illustrated in Figure 4, which plots potential energy as a function of the reaction coordinate. The “dark” state is populated from the FC state in a sub-picosecond process in 1GM and in <50 fs (or directly) in 1GM_CT_, the difference suggesting different initial ground state structures or a steeper excited state potential in 1GM_CT_. Such a substituent-dependent ground state structure is suggested by the non-resonant Raman spectra of 1GM and 1GM_CT_ and the DFT calculations, both of which show different vibrational spectra for the two motors (see Figure S15). The most obvious spectral differences are observed in 1550–1650 cm^–1^ (corresponding to the C=C stretching frequency of the ring and axle), in 1200–1475 cm^–1^ (corresponding to the ring breathing modes), and in an enhanced mode at 520 cm^–1^ (assigned by DFT to a pyramidalization plus ring deformation coordinate; Supporting Information). The DFT optimized structures also show substituent-dependent changes in the C=C axle bond length as well as the dihedral angle around the axle (see Table S1). These ground state structure changes involve the torsion and pyramidalization coordinates, which are shown to be critical in the excited state reaction by quantum chemical calculations.^24−26^ This is consistent with the observed perturbation to the FC state.

The dark state has a picosecond lifetime, but there is no correlation between this lifetime and the isomerization yield, suggesting that the two are independent. This is assigned to the presence of a low barrier between the dark state and the conical intersection connecting the excited state to the ground state, with the rate of barrier crossing determining the dark state decay time, but not the yield of the metastable isomer. The lifetime for 1GM is consistently longer than for 1GM_CT_, showing that the CT substituent has lowered the barrier to the CI(s). For both 1GM and 1GM_CT_, the shorter lifetime is observed in methanol, indicating that the barrier height is decreased in polar solvents (these observations are also consistent with emission yield data; Table S2). After barrier crossing, the excited state relaxes to the ground state without any further intermediate being identified in TA, meaning that the reaction proceeds directly through the CI on a subpicosecond time scale. We suggest that the dramatic effect of the CT substituents on the photochemical yield (Table 1) arises from a change in the location and topography of the CI (Figure 4). The enhanced yield in the nonpolar solvent (Table 1) is ascribed to the substituents shifting the location of the CI to a geometry where it more closely reflects the structure of the metastable photoproduct. The dramatic solvent polarity effect could be introduced either by a solvent-dependent shape of the excited state potential surface between the dark state and CI, shifting the CI closer to the reactant geometry in the polar solvent, or to a solvent-dependent change in CI topography. In the latter case, the topography would change from one favoring the product (say a peaked CI) in the nonpolar solvent to one favoring the initial reactant isomer (e.g., a sloped CI) in the polar solvent. Again, this could arise from a solvent dependence of the potential energy surface. Significantly, there is some precedent for these possibilities in calculations. Filatov and Olivucci showed that breaking the symmetry around a double bond in an isomerization reaction by the introduction of an electron withdrawing group in conjugation with that bond stabilizes a CT configuration without the need for pyramidalization, thus leading to a change in the location of the CI.^23^ Further, Malhado and Hynes have shown how the peaked or sloped topology of a CI can be modified by dynamical solvent effects.^32^ Further understanding of the details of how CT substituents modify the isomerization yield in molecular motors requires detailed quantum chemical calculations of the effect of CT substituents on motor dynamics alongside additional experimental measurements with different substituents.

## Conclusions

Donor and acceptor substituents were placed in conjugation with the double bond that dominates the photochemical reaction coordinate in overcrowded alkene photomolecular motors. This was shown to modify the yield of the photochemical isomerization in a solvent polarity-dependent fashion. The origin of the effect was discussed in terms of an increased weight of a CT configuration modifying the excited state reaction coordinate and leading to solvent polarity-dependent changes in the CI with the ground state (Figure 4). Given that the excited state reactions of second- and third-generation motors have similar potential surfaces and ultrafast dynamics to those of 1GM, we anticipate that a similar approach will modify their efficiencies as well. This is significant as those motors have overall lower yields for metastable state formation, which might be similarly enhanced by CT substituents. Specifically in the context of 1GMs, it would be interesting to investigate whether the second (trans stable to cis stable; Figure 1a) half of the photocycle would be similarly sensitive to CT substituents. The TAs of 1GM in the stable cis and trans ground states were measured by Wiley et al.^19^ The yields were similar as were the excited state decay pathways. However, there were subtle differences in decay times and polarity effects. The similarities suggest that CT substituents would add a control dimension in this reaction too, but further experiments are required to test this. Further, the key role of solvent polarity for 1GM_CT_ demonstrated here adds an important new degree of freedom in the potential exploitation of push–pull molecular motors. For example, in life science applications, it offers the possibility of selecting a motor for its efficiency in a particular environment, which has implications for phototherapeutics among others. Future work will investigate the extent to which the electron withdrawing/donating capacity of the substituents provides fine control over motor efficiency in first- and later-generation motors.

## Experimental Methods

The synthesis of 1GM was described elsewhere.^33^ 1GM_CT_ was synthesized via mixed McMurry reaction following the procedure detailed in the Supporting Information. Full details along with the characterization are given in Figures S1–S13. Isomerization quantum yields were determined using an in situ irradiation NMR method, also described in detail in the Supporting Information. Methods for broadband transient absorption are described elsewhere^34^ and are further detailed in the Supporting Information.

## Supporting Information Available

Full experimental details concerning the synthesis and measurements, additional data on fluorescence quantum yields, the transient absorption of other 1GM derivatives, and time-resolved fluorescence (PDF)
