# Supplementary material for: Control of Photoconversion Yield in Unidirectional Photomolecular Motors by Push–Pull Substituents
Source: J Am Chem Soc. 2023 Aug 30;145(36):19849–55. doi: 10.1021/jacs.3c06070 (PMC10510317; doi:10.1021/jacs.3c06070)
Supplement: Supplementary file 1 — ja3c06070_si_001.pdf [file ja3c06070_si_001.pdf]

SUPPLEMENTARY INFORMATION

Control of Photoconversion Yield in Unidirectional Photomolecular Motors by Push-Pull Substituents

Palas Roy,[1,4] Andy S. Sardjan,[2] Wojciech Danowski,[2,5] Wesley R. Browne,[3] Ben L. Feringa[2]* and Stephen R. Meech[1]*

[1]*School of Chemistry, University of East Anglia, Norwich NR4 7TJ, U.K.*

[2]*Centre for Systems Chemistry, Stratingh Institute for Chemistry, University of Groningen, 9747AG Groningen, The Netherlands*

[3]*Molecular Inorganic Chemistry, Stratingh Institute for Chemistry, University of Groningen, 9747AG Groningen, The Netherlands*

[4]*School of Basic Sciences, Indian Institute of Technology Bhubaneswar, Odisha 752050, India*

[5]*University of Strasbourg, CNRS, ISIS UMR 7006, 8 allée Gaspard Monge, F-67000 Strasbourg, France.*

Experimental Details

Synthesis and Characterisation:

All reagents were obtained from commercial sources and used as received without further purification. Dry solvents were obtained from an MBraun solvent purification system. Column chromatography was performed on a Reveleris X2 flash chromatography system. TLC: silica gel 60, Merck, 0.25 mm. High resolution mass spectrometry (ESI or APCI-MS) was performed on a LTQ Orbitrap XL spectrometer with ESI or APCI ionization. NMR spectra were recorded on Varian AMX400 (^{1}H: 400 MHz, ^{13}C: 101 MHz) and Varian Unity Plus (^{1}H: 500 MHz, ^{13}C: 126 MHz, ^{19}F: 471 MHz) spectrometers. Chemical shifts are reported in parts per million (ppm) relative to the residual solvent signal (for $CDCl_3$ δ 7.26 for ^{1}H, δ 77.2 for ^{13}C and for C_6D_6 δ 7.16 for ^{1}H, δ 128.1 for ^{13}C). ^{19}F spectra were recorded without reference and are uncorrected. For ^{1}H NMR spectroscopy, the splitting pattern of peaks is designated as follows: s (singlet), d (doublet), t (triplet), m (multiplet), br (broad), or dd (doublet of doublets). Indanones[1-2] were synthesized according to procedure reported in the literature.

For all the motors NMR spectra were recorded in $CDCl_3$ passed through a K_2CO_3 plug. Any traces of acidic impurities in $CDCl_3$ were found to decompose the motors. Likewise stray ambient light was found to induce isomerization of the motors; hence all the reactions were carried in the dark by protecting the flasks in tin foil and all the manipulations were carried in the dark. Likewise, NMR samples were stored in the dark and protected from stray light before measurements.

For the safety reason, all the reaction carried with $Zn(CN)_2$ should be performed at maximum 500 mg scale of $Zn(CN)_2$ with uttermost care. Zinc cyanide should be weighted under well-ventilated fume hood in a closed vial to avoid contamination of the common balance area. All the aqueous wastes should be stored in an appropriately labelled container in basic pH and disposed appropriately. All the glassware and spatulas should be rinsed with basic pH water. All the gloves, septa, or plastic caps contaminated with $Zn(CN)_2$ should be stored in a separate waste container and disposed appropriately. See the links for further information regarding handling and before the use: https://www.uwindsor.ca/chemical-control-centre/sites/uwindsor.ca.chemical-control-centre/files/ccc-2012_cyanide_storage_handling_and_general_information_1.pdf or http://www.cchem.berkeley.edu/rsgrp/SOPs/new/Zinc%20Cyanide_Sarpong_Final.docx

Isomerisation quantum yield measurements:

A general method was adopted from (3).

An optical fibre was prepared by cutting off one end, stripping all the insulating materials and etching the final cladding with sandpaper. This fibre was then inserted and glued to a wilmad CFQ 2 mm diameter EPR tube. This fibre was than attached to an NMR tube cap with a hole drilled in to ensure the sample fibre height in all samples.

Actinometry was done using ortho-nitrobenzaldehyde with concentration of 20 mg/mL in CD_2Cl_2 and motor kinetic runs were done at high enough concentration for total absorbance. The sample was irradiated in the NMR for at least 20 min to get the initial rate of change of concentration. In general the rate of change of concentration of a PT type photo switch is given by:

$$\frac{d[A]}{dt} = -\frac{I_0}{V}\frac{D_A}{D_{tot}}\left(1-10^{-D_{tot}}\right)\Phi_f + \frac{I_0}{V}\frac{D_B}{D_{tot}}\left(1-10^{-D_{tot}}\right)\Phi_r + k[B]$$

However if we only have [A] present at the start and we have total absorbance of the light, then the initial rate will be given by:

$$\left.\frac{d[A]}{dt}\right|_{\text{initial}} = \frac{I_0}{V}\Phi_A$$

This measurement is done with actinometer and with motor, both under total absorbance conditions, using the decay of a peak divided by the number of protons it represents in the molecule, then the quantum yield for the switching of the motor is given by:

$$\Phi = \frac{\left.\frac{d[motor]}{dt}\right|_{\text{initial}}}{\left.\frac{d[act]}{dt}\right|_{\text{initial}}} * \Phi_{act}$$

Both rates are obtained by dividing the slope of the integrals over time by the number of protons that represent it. The known quantum yield of ortho-nitrobenzaldehyde at 365 nm is 0.5.[4] Both rates were measured at least twice to show reproducibility of sample tube and fibre.

The used light source was a 365 nm LED, M365FP1 from Thorlabs and the fibre was a custom 4 m high OH fibre.

For all ^{1}H-NMR's the following settings were used: delay time of 5 s, 8 scans averaging and 4 steady state scans before each spectrum in the kinetic run.

**Compound**	**Solvent**	**Rate corrected for num protons**	**Known Quantum Yield**	**Calculated Quantum Yield**
**ONB**	CD_2Cl_2	0.176	0.5	
**1GM**	$CDCl_3$	0.239		0.68
**1GM_CT**	$CDCl_3$	0.0149		0.042
**ONB**	CD_2Cl_2	0.180	0.5	
**1GM**	CD_3OD	0.152		0.42
**1GM_CT**	CD_3OD	0.0181		**0.0**50
**ONB**	CD_2Cl_2	0.172	0.5	
**1GM**	C_6D_{12}	0.220		0.64
**1GM_CT**	C_6D_{12}	0.342		0.99

The actinometry was performed before and after the quantum yield determination and averaged. All different solvent measurements were done on different days so the actinometry was repeated.

Steady-state absorption: Steady-state absorption spectra of motors were recorded using a PerkinElmer Lambda XLS spectrophotometer. Quartz cuvette with path length of 1 cm was used.

Steady-state fluorescence: Steady-state Fluorescence spectra of motors in different solvents were recorded using the Edinburgh Instruments FS5 Fluorimeter . The excitation wavelength used was 380 nm and 400 nm for 1GM and $1GM_{CT}$, respectively. 1 cm path length cuvette was used for all the measurements while keeping sample absorbance below 0.2 OD.

In order to avoid build up and emission from the metastable and trans isomers the excitation bandwidth was kept below 0.2 nm. Many spectra were recorded and averaged before any changes in the spectra could be observed.

Time-resolved Fluorescence Up-Conversion: The detailed description of the time-resolved fluorescence set up was reported elsewhere.[5] Briefly, the femtosecond mode locked Ti:Sapphire laser oscillator (Coherent Micra-10) produced 20 fs pulses centred at 800 nm at a 76 MHz repetition rate and a power of 920 mW. The output from the oscillator was passed through a fused silica prism compressor and then focused onto a 50 µm thick type I BBO crystal with a 150 mm focal length concave mirror. This produces up to 15 mW of 400 nm second harmonic excitation pump beam. A dichroic mirror was used to separate the pump (second harmonic) and fundamental (which was used as the Gate pulse) beams. A pair of chirped mirrors (Femtolasers GSM216) was used to compress the gate beam (where the dispersion is introduced by the dichroic mirror and the focusing lens). The gate beam is then directed through an optical delay line (Physik Instrumente). Another pair of chirped mirrors (Femtolasers GSM012) is used to compress the 400 nm pump beam (where the dispersion is introduced by the sample cell window). The pump beam was focused at the sample cell of 1 mm pathlength with a concave mirror of focal length 150 mm. The fluorescence generated from the sample was collected and focused onto a BBO crystal using a reflective microscope objective (with 15 times magnification and a back focal length of 160 mm). Then the fluorescence and gate (800 nm) beams were focused and mixed in a 100 µm type I BBO crystal to generate up-converted light. The up-converted light was detected by a photomultiplier and monochromator combination and measured with a photon counter. A filter (GG455, Schott) after the objective (before the up-converting BBO crystal) and another filter (UG11, Schott) at the entrance slit of the monochromator were placed to isolated the up-converted signal from the intense pump scattering. Upconversion signal of Raman scattering from heptane at 470 nm was used to determine the IRF and time zero for the experiment which is found to be around 43 fs. Photo-damage was minimised by flowing the solution (using a peristaltic pump) and using pump beam power of 5 mW (focal spot of ca 200 µm inside cuvette). Sufficient volume (150 mL) of the solution was used to ensure that build-up of the metastable form was negligible. The kinetics analyses were carried out using IGOR pro 5 wavemetrics software to determine decay time constants and their respective amplitudes.

Femtosecond transient absorption measurements (TA): The detailed description of transient absorption set up used here has been described elsewhere.[6] Briefly TA is a two pulse technique where a femtosecond pump pulse electronically excites the molecules and then a time delayed broadband white light continuum probe pulse captures the resultant changes in the electronic

spectra. The fundamental beam from the Spectra Physics Mai Tai laser oscillator is amplified using a regenerative Ti:sapphire amplifier (Spectra Physics Spitfire ACE). The amplified output pulses centered at 800 nm (with duration of 120 fs, repetition rate of 1 kHz and energy of 5 mJ per pulse) are then used to drive two commercial optical parametric amplifiers (OPA, Light Conversion TOPAS Prime). One OPA generates the actinic pump pulse (with duration of 80 fs) which is used to excite the motor samples. The pump pulse was then passed through a mechanical chopper at 500 Hz and a computer-controlled delay stage. A combination of half wave plate and a polarizer in the pump path was used to set polarization at magic angle. The second OPA was tuned to generate the 1250 nm pulse which was then focused on to a 3 mm thick sapphire window to generate broadband white light continuum in the 500-1400 nm spectral windows. The fundamental beam at 800 nm was directly focused onto the sapphire plate to access the WLC probe in the 370-800 nm spectral windows. All the TA Spectra were first recorded in two separate overlapping WLC probe windows and then stitched together.

The WLC beam was split using a 50/50 beam splitter before the sample stage. One part (the Reference WLC) was detected in the reference detection channel to correct for intensity fluctuations in the WLC probe spectrum. Another part (the Probe WLC) was spatially and temporally overlapped with the actinic pump pulse in a 1 mm thick sample cell (with 0.5 mm thick fused silica windows). The focal spot sizes of the pump and probe pulses were adjusted at 250 µm and 50 µm respectively. The pump beam power at the sample cell was attenuated to 0.2 mW. The probe beam was aligned collinearly with the reference beam and on top of each other after passing through the sample stage. A home-built prism based spectrograph was used to disperse both the WLC beams which were then focused and detected by two separate synchronised 16 bit A/D CCD detectors (from Entwicklungsbüro Stresing detector, 1024 pixels). The chopper at 500Hz in the pump beam path allowed the detection of pump-on/pump-off spectra in both the probe and reference windows. The referenced difference spectrum (ΔA) between pump-on and pump-off was calculated using:

$$\Delta A = -log(\frac{Probe\ (Pump\ on)\ \mathrm{X}\ Refernce\ (Pump\ off)}{Reference\ (Pump\ on)\ \mathrm{X}\ Probe\ (Pump\ off)})$$

The detector was calibrated using a Mercury-Argon lamp (HG-1 Ocean Optics) or a calibrated filter. The instrument response function (IRF) which was determined using the solvent Kerr response and was found to be about 100 fs. The data presented were average over 3 cycles and each time trace was accumulated for 0.5 sec. All the TA measurements were done under flowing condition in a 1 mm quartz cell with the liquid driven by a peristaltic pump (World Precision Instruments). The sufficient

volume (150 mL) of the solution was used to ensure that build-up of the metastable form was negligible.

Global Analysis: Glotaran 1.5.1 software package[7] was used to analyse the TA dataset. Global analysis was performed to report both the Evolution Associated Difference Spectra (EADS) and lifetimes associated with each species. Here we used a sequential model for transition from bright state to the metastable product via excited state intermediates.

Steady-state Raman. Steady-state non-resonant Raman spectra of the motors were recorded using excitation laser at 532 nm (25 mW, Cobolt lasers) equipped with Olympus BX51 upright microscope dichroic filter at 45 degrees. A MM fibre optic was used to collect and guide the Raman scattering to a Shamrock300i spectrograph and Newton EMCCD-BU (Andor Technology). The Raman data was baseline corrected using Spectragryph.

Quantum yields of Fluorescence. Emission quantum yields were measured using the FS5 Edinburgh instruments Fluorimeter, using the SC-30 integration sphere. Briefly the measurement uses spectra recorded of sample and solvent as a reference, while scanning over the excitation band. The excited band intensity is decreased due to the sample (motor) absorbing some of the light. Since the spectrometer is corrected, the decrease in intensity versus the emission band can be used to calculated the absolute emission quantum yield. This was performed for the motor in methanol and cyclohexane. The yields are given in Table S2.

DFT Calculation. Density Functional Theory (DFT) calculations for Raman spectra of molecular motors have been carried out at the rb3lyp/tzvp level using Gaussian16 program.[8] The motor structure was first optimized (see Table of atomic coordinates) and then Raman spectra were calculated (as shown by red vertical lines in SI Figure S3). To account for a systematic error in the DFT predictions, all the frequencies have been scaled by a factor of 0.98.

Synthesis

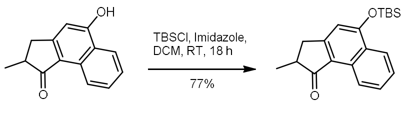

S1 S2

5-((tert-butyldimethylsilyl)oxy)-2-methyl-2,3-dihydro-1H-cyclopenta[a]naphthalen-1-one (S2)

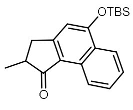

To a solution of[1] 5.0 g (23.6 mmol, 1.0 equiv.) in CH_2Cl_2 (100 mL) was added imidazole 2.3 g (33.0 mmol, 1.4 equiv.) and tertbutyldimethylsilyl chloride 4.6 g (30.6 mmol,

1.3 equiv.). Upon stirring for 30 mins. a precipitate was formed, and the reaction mixture was stirred for 18 h at RT. The solution was washed with HCl, water, saturated aqueous $NaHCO^3$ and brine. The organic phase was dried over $MgSO_4$ and concentrated in vacuo. The crude product was purified by column chromatography (SiO_2, pentane/EtOAc) to afford 5.9 g (18.1 mmol, 77%) of **S2** as a waxy white solid

^{1}H NMR (400 MHz, $CDCl_3$) δ 9.14 (dt, J = 8.4, 1.0 Hz, 1H), 8.26 – 8.17 (m, 1H), 7.66 (ddd, J = 8.3, 6.9, 1.3 Hz, 1H), 7.53 (ddd, J = 8.3, 6.9, 1.3 Hz, 1H), 6.80 (s, 1H), 3.48 – 3.24 (m, 1H), 2.87 – 2.68 (m, 2H), 1.36 (d, J = 7.3 Hz, 3H), 1.11 (s, 9H), 0.36 (s, 6H).

^{13}C NMR (101 MHz, $CDCl_3$) δ 208.6, 158.9, 158.8, 131.3, 129.4, 127.5, 126.0, 124.2, 124.1, 123.2, 109.9, 42.4, 35.8, 25.9, 18.6, 17.0, -4.0.

HRMS (ESI+) $[M+H]^+$ calcd for $C_{20}H_{26}O_2SiH^+$ 327.1775, found 327.1778.

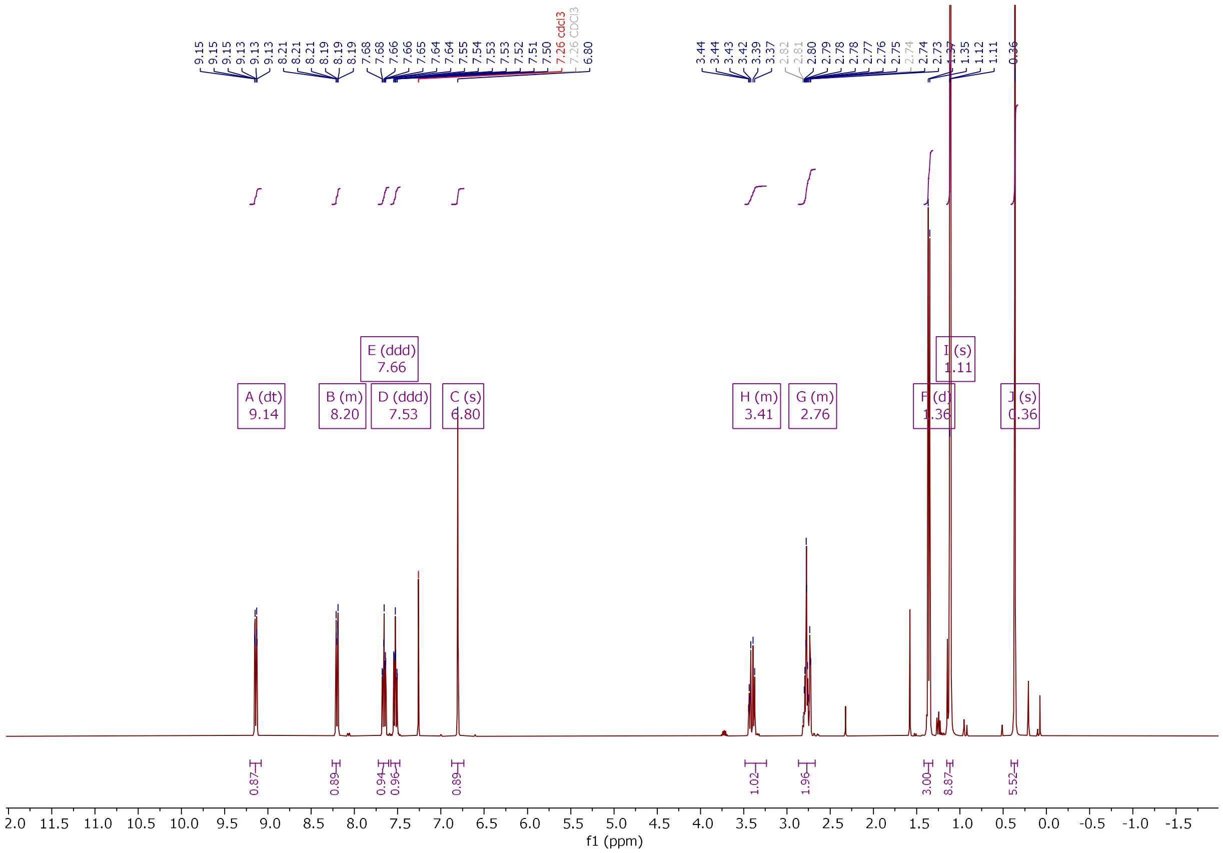

Figure S1 ^{1}H NMR (400 MHz, $CDCl_3$, RT) spectrum of **S2**

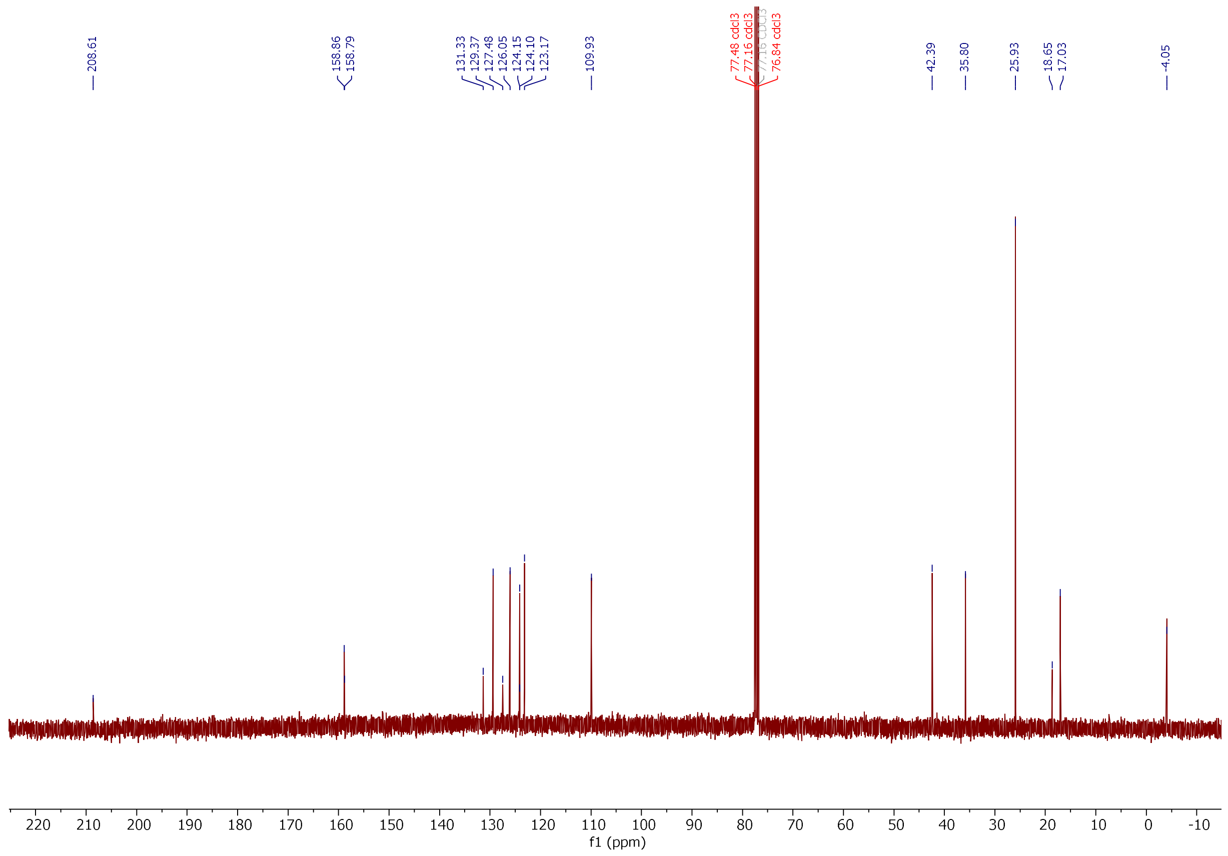

Figure S2 ^{13}C NMR (101 MHz, $CDCl_3$, RT) spectrum of **S2**

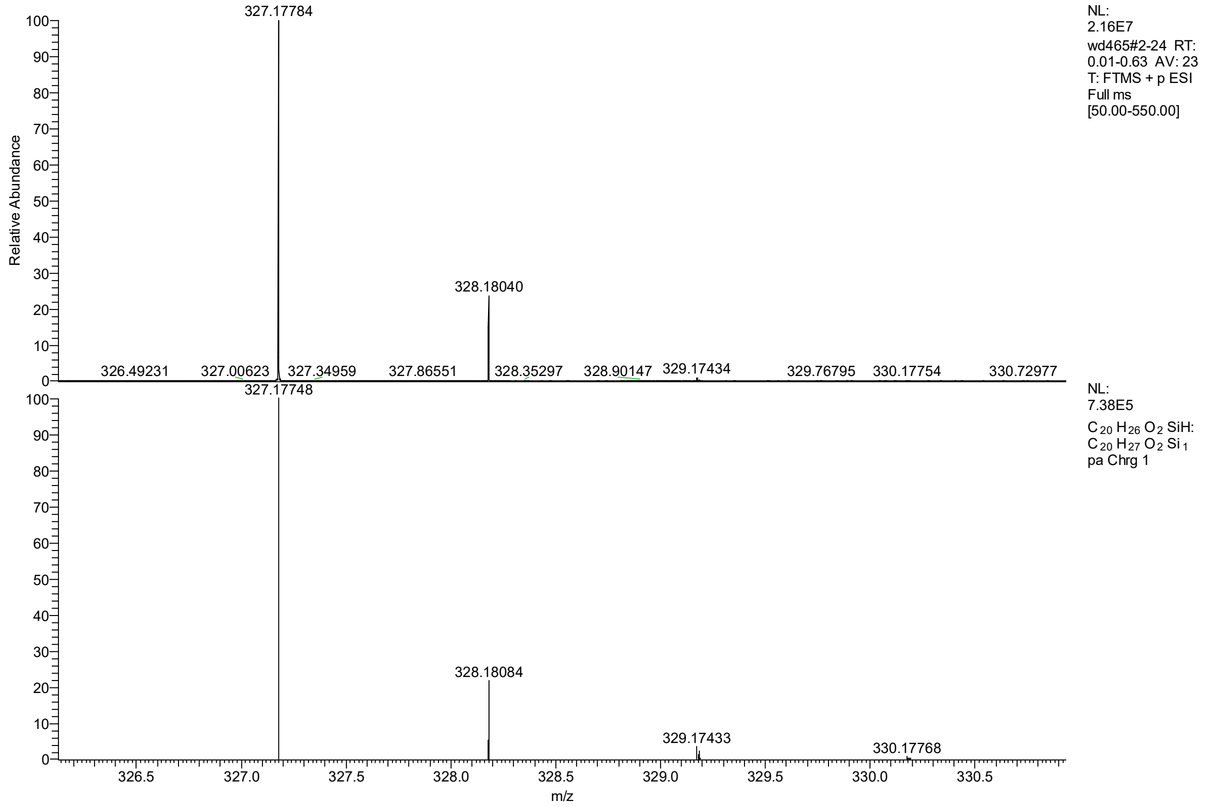

Figure S3 HRMS spectrum of (ESI+) compound **S2** (top: measured, bottom: calculated).

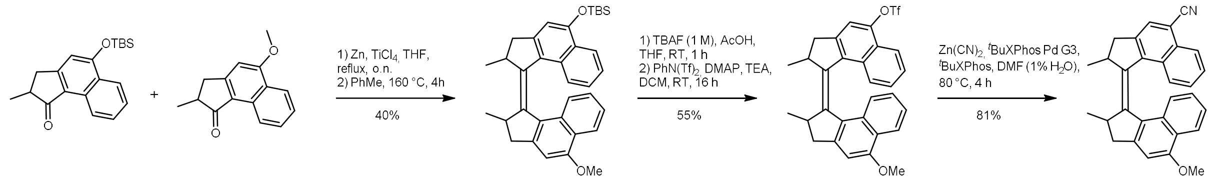

S2 S3 S4 S5 1GM$_{CT}$

(*Z*)-tert-butyl((5'-methoxy-2,2'-dimethyl-2,2',3,3'-tetrahydro-[1,1'-bi(cyclopenta[a]naphthalenylidene)]-5-yl)oxy)dimethylsilane (S4)

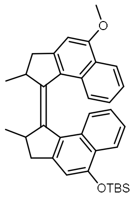

Under nitrogen atmosphere, to a suspension of Zn powder (6.01 g, 91.9 mmol, 12.0 equiv.) in 40 mL of anhydrous THF cooled to 0 °C a $TiCl_4$ (5.00 mL, 45.9 mmol, 6.0 equiv.)was slowly added via a syringe. The reaction mixture was heated at reflux for 3 h and cooled to RT. Next, a solution of 5-((tert-butyldimethylsilyl)oxy)-2-methyl-2,3-dihydro-1H-cyclopenta[a]naphthalen-1-one (**S2**) (2.50 g, 7.66 mmol, 1.0 equiv.) and 6-methoxy-2,4,7-trimethyl-2,3-dihydro-1H-inden-1-one[2] (**S3**) (3.47 g, 15.3 mmol, 2.0 equiv.) in THF (20 mL) (suspension should be heated gently to fully dissolve the compounds) was added via syringe. The mixture was heated at reflux for 24 h. After cooling to room temperature, the reaction mixture was poured onto a mixture of aqueous HCl (1 M, 200 mL) and crushed ice and extracted with EtOAc (3 x 500 mL). The organic layer was washed with water, saturated aqueous solution of $NaHCO_3$ (200 mL), brine (300 mL) and dried over Na_2SO_4. The solvents were evaporated under reduced pressure and crude was filtrated through a short silica pad (pentane/DCM 1:1 *v*/*v*). Filtrate was concentrated under reduced pressure after which the solid residue was suspended in toluene (20 mL) and the mixture was heated at 160 °C in a sealed tube for 4 h to induce thermal *E*/*Z* isomerization of the alkenes. The crude product was purified by flash column chromatography (SiO_2, pentane/DCM) and crystalized from EtOH/DCM yielding 1.61 g (3.09 mmol, 40%) of **S4** as yellow crystals.

^{1}H NMR (500 MHz, $CDCl_3$) δ 8.09 – 8.02 (m, 1H), 7.97 (d, J = 8.3 Hz, 1H), 7.00 – 6.90 (m, 2H), 6.88 (d, J = 15.6 Hz, 2H), 6.66 (t, J = 9.0 Hz, 2H), 6.40 (dddd, J = 8.3, 6.8, 3.7, 1.3 Hz, 2H), 4.05 (s, 3H), 3.63 – 3.45 (m, 4H), 2.66 – 2.52 (m, 2H), 1.20 (d, J = 6.2 Hz, 6H), 1.11 (s, 9H), 0.37 – 0.28 (d, 6H). (Resonances corresponding to protons of two diastereotopic methyl groups in TBS were observed as apparent doublet)[9].

^{13}C NMR (126 MHz, $CDCl_3$) δ 155.7, 151.9, 144.6, 144.4, 137.9, 137.7, 131.1, 130.7, 130.7, 129.7, 126.9, 126.9, 126.4, 124.6, 124.5, 124.1, 123.6, 123.4, 122.4, 121.6, 111.1, 102.2, 55.7, 42.0, 42.0, 41.4, 41.0, 26.1, 21.2, 21.2, 18.7, -3.9, -4.0. (Two resonances were observed for diastereotopic methyl carbons of TBS group)[9].

HRMS (ESI+) $[M]^+$ calcd for $C_{35}H_{40}O_2Si^+$ 520.2792, found 520.2783.

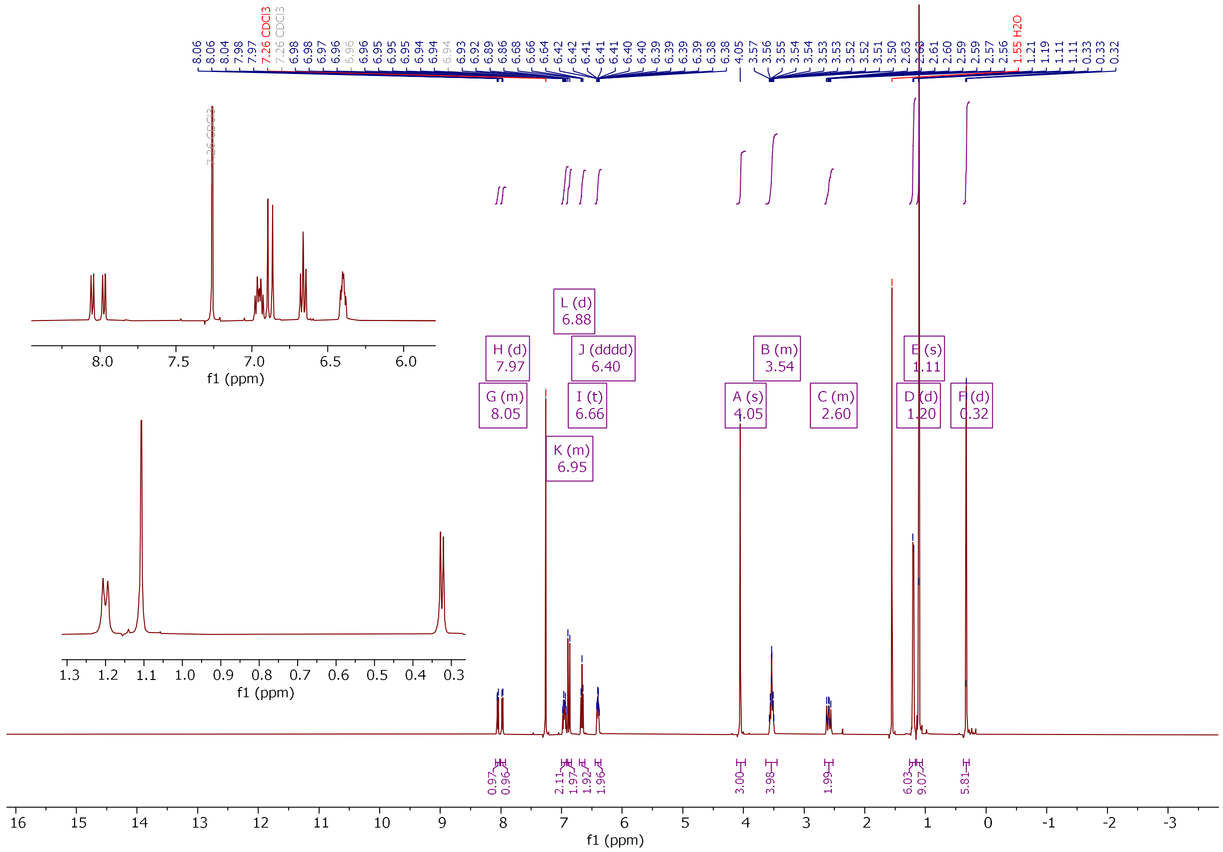

Figure S4 ^{1}H NMR (500 MHz, $CDCl_3$, RT) spectrum of **S4**

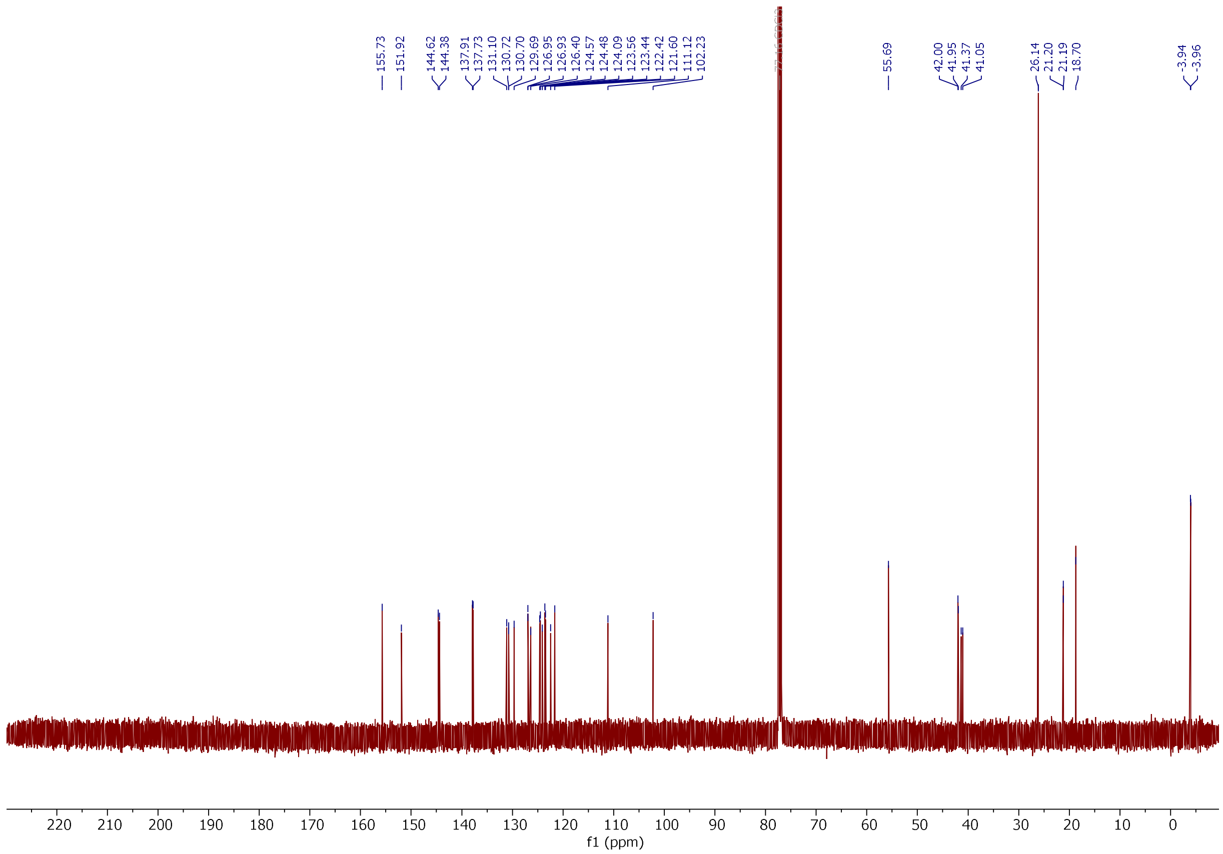

Figure S5 ^{13}C NMR (126 MHz, $CDCl_3$, RT) spectrum of **S4**

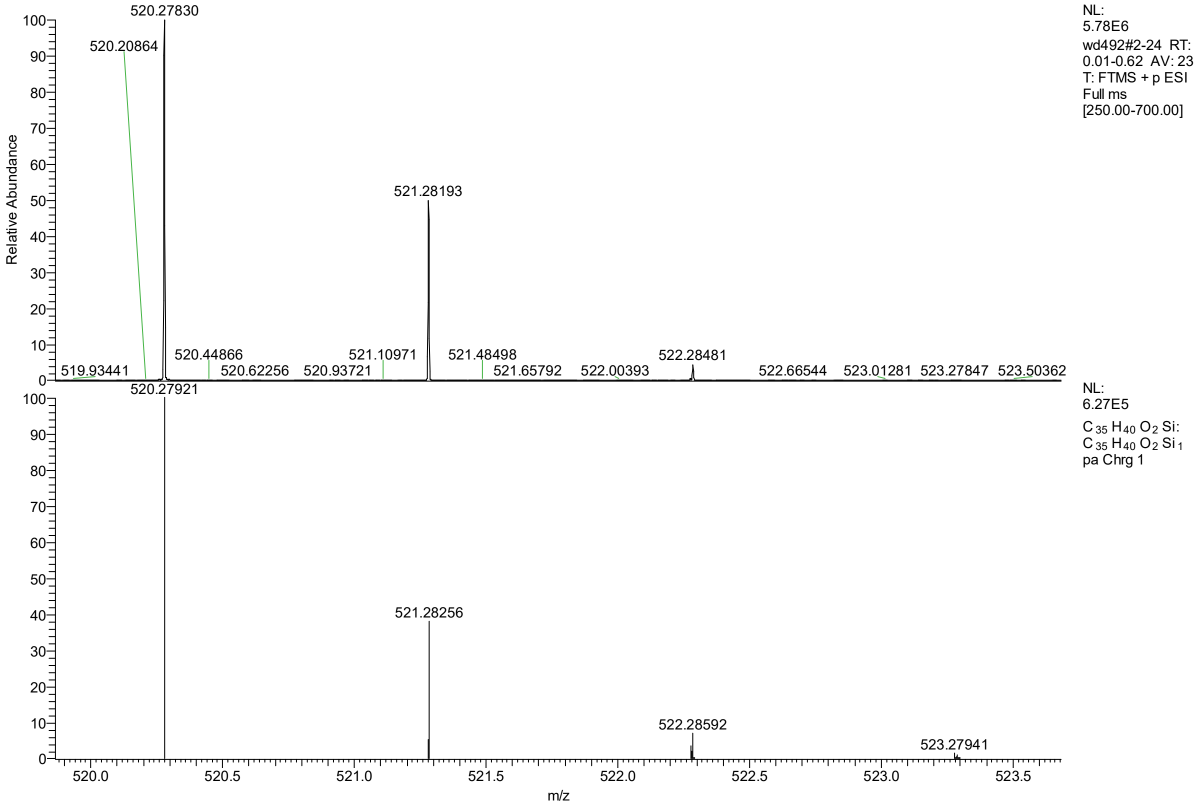

Figure S6 HRMS spectrum of (ESI+) compound **S4** (top: measured, bottom: calculated).

(Z)-5'-methoxy-2,2'-dimethyl-2,2',3,3'-tetrahydro-[1,1'-bi(cyclopenta[a]naphthalenylidene)]-5-yl trifluoromethanesulfonate (**S5**)

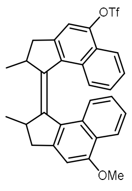

Under nitrogen atmosphere, to a solution of **S4** (1.56 g, 3.00 mmol, 1.0 equiv.)in 40 mL THF and 15 mL of AcOH a solution of TBAF (1 M in THF, 15.0 mL, 15.0 mmol, 5.0 equiv.) was added via a syringe and the mixture was stirred at RT for 1 h. The mixture was diluted with EtOAc and washed copiously with water, brine and dried over Na_2SO_4 and concentrated *in vacuo*. The deprotected motor was found to readily decompose under ambient conditions therefore it was used directly in the next step without further purification or analysis. Next, the resulting solid was dissolved in DCM (30 mL) and N-Phenyl-bis(trifluoromethanesulfonimide) ($PhN(Tf)_2$) (1.39 g, 3.89 mmol) 1.3 equiv.), DMAP (30 mg, 300 µmol, 0.1 equiv.) and triethyl amine (TEA) (2.55 mL, 18.3 mmol, 1.5 equiv.) were added. The resulting suspension was stirred at RT for 16 h upon which the solids dissolved. Next the mixture was diluted with DCM, washed with 10% aqueous HCl (30 mL), times with water, saturated aqueous $NaHCO_3$, brine dried over Na_2SO_4, filtrated, and concentrated *in vacuo*. The crude product was purified by flash column chromatography (SiO_2, pentane/EtOAc) yielding 0.81 g (1.50 mmol, 50%) of **S5** as yellow solid.

^{1}H NMR (500 MHz, $CDCl_3$) δ 8.13 – 8.00 (m, 1H), 7.84 (d, J = 8.5 Hz, 1H), 7.47 (s, 1H), 7.12 – 7.06 (m, 1H), 7.00 – 6.93 (m, 1H), 6.87 (s, 1H), 6.74 (d, J = 8.5 Hz, 1H), 6.51 – 6.45 (m, 1H), 6.45 – 6.34 (m, 2H), 4.06 (s, 3H), 3.72 – 3.47 (m, 4H), 2.75 – 2.60 (m, 2H), 1.29 – 1.16 (m, 6H).

^{13}C NMR (126 MHz, $CDCl_3$) δ 156.4, 145.7, 145.0, 142.8, 142.1, 138.3, 135.9, 130.6, 130.4, 128.6, 127.1, 126.5, 126.1, 125.4, 125.0, 124.8, 124.1, 123.9, 121.9, 120.5, 120.2 (OTf), 117.4 (OTf), 115.7, 102.2, 55.7, 42.1, 42.1, 41.3, 40.7, 21.1, 20.9. (Only two resonances were observed for the CF_3 quartet)

^{19}F NMR (471 MHz, $CDCl_3$) δ -73.27.

HRMS (APCI+) $[M+H]^+$ calcd for $C_{30}H_{26}F_3O_4S^+$ 539.1498, found 539.1498.

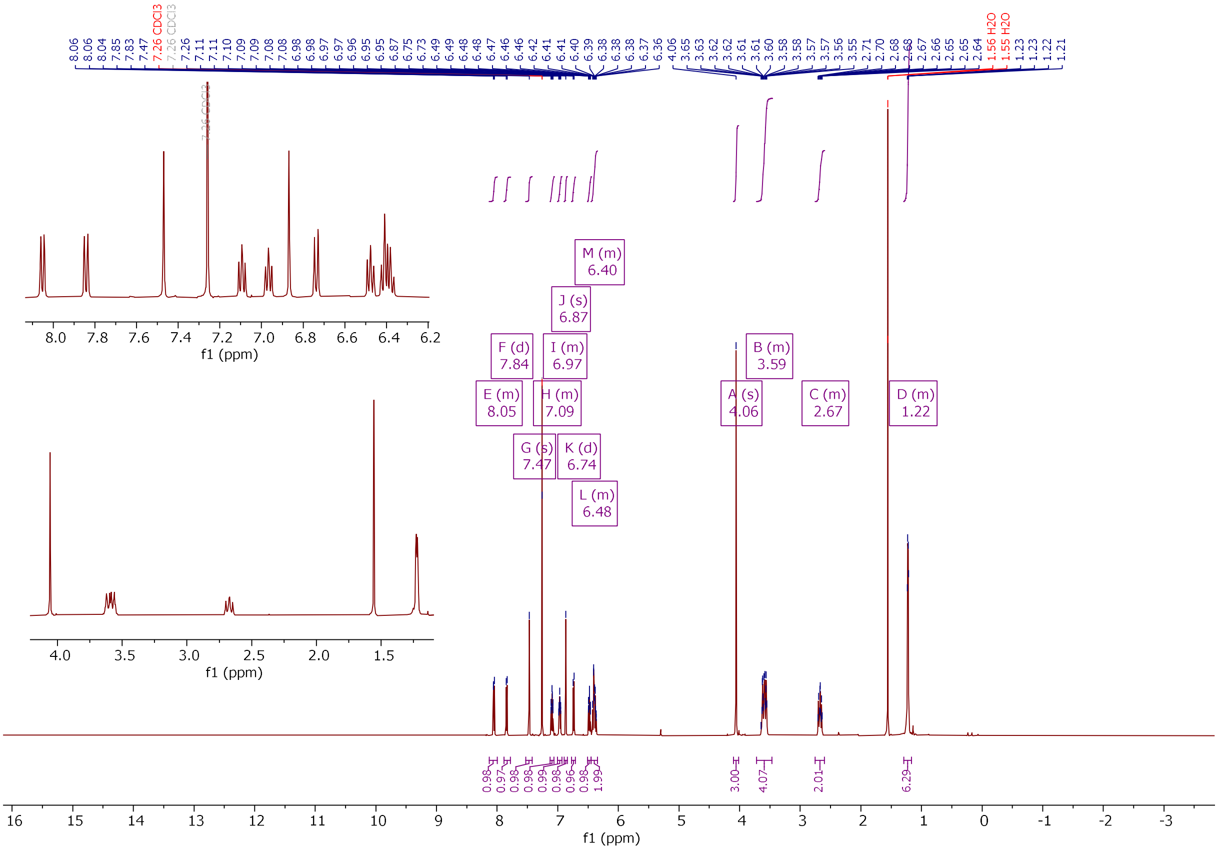

Figure S7 ^{1}H NMR (500 MHz, $CDCl_3$, RT) spectrum of **S5**

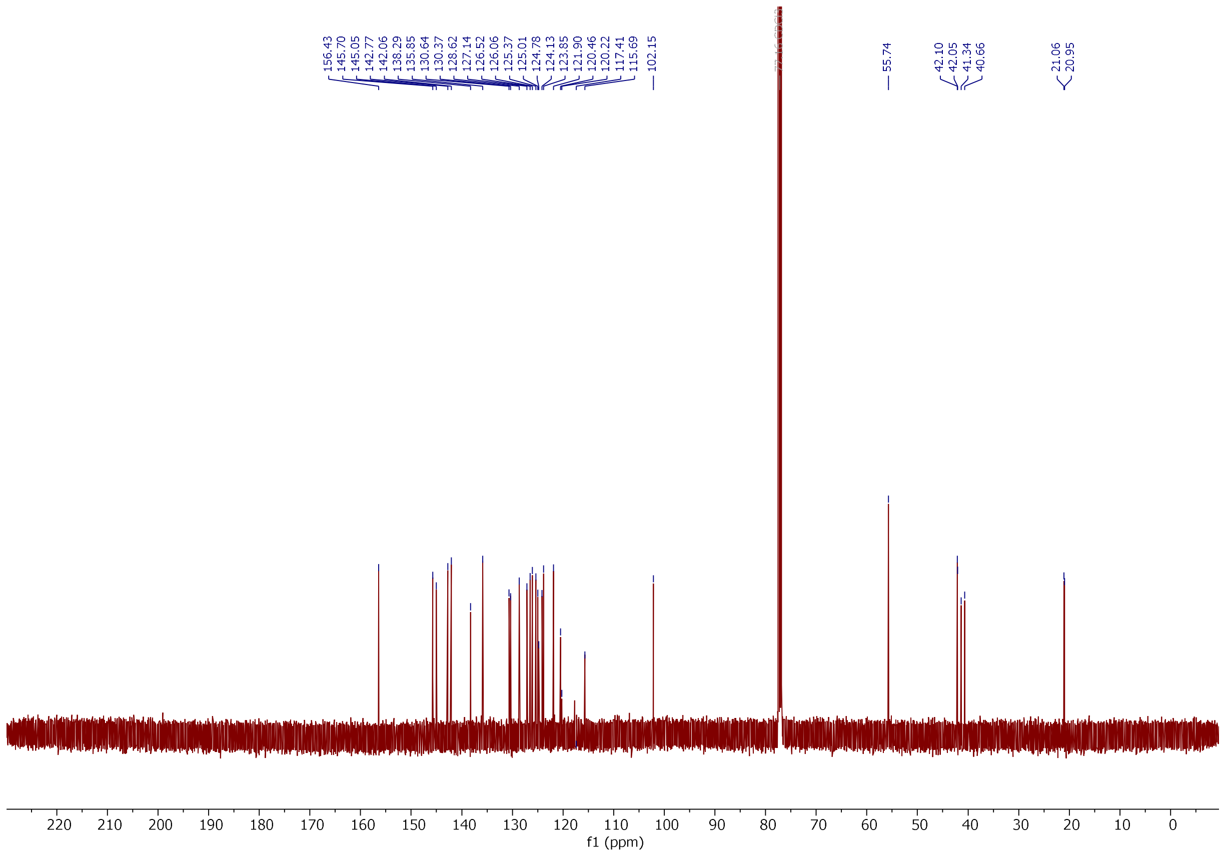

Figure S8 ^{13}C NMR (126 MHz, $CDCl_3$, RT) spectrum of **S5**

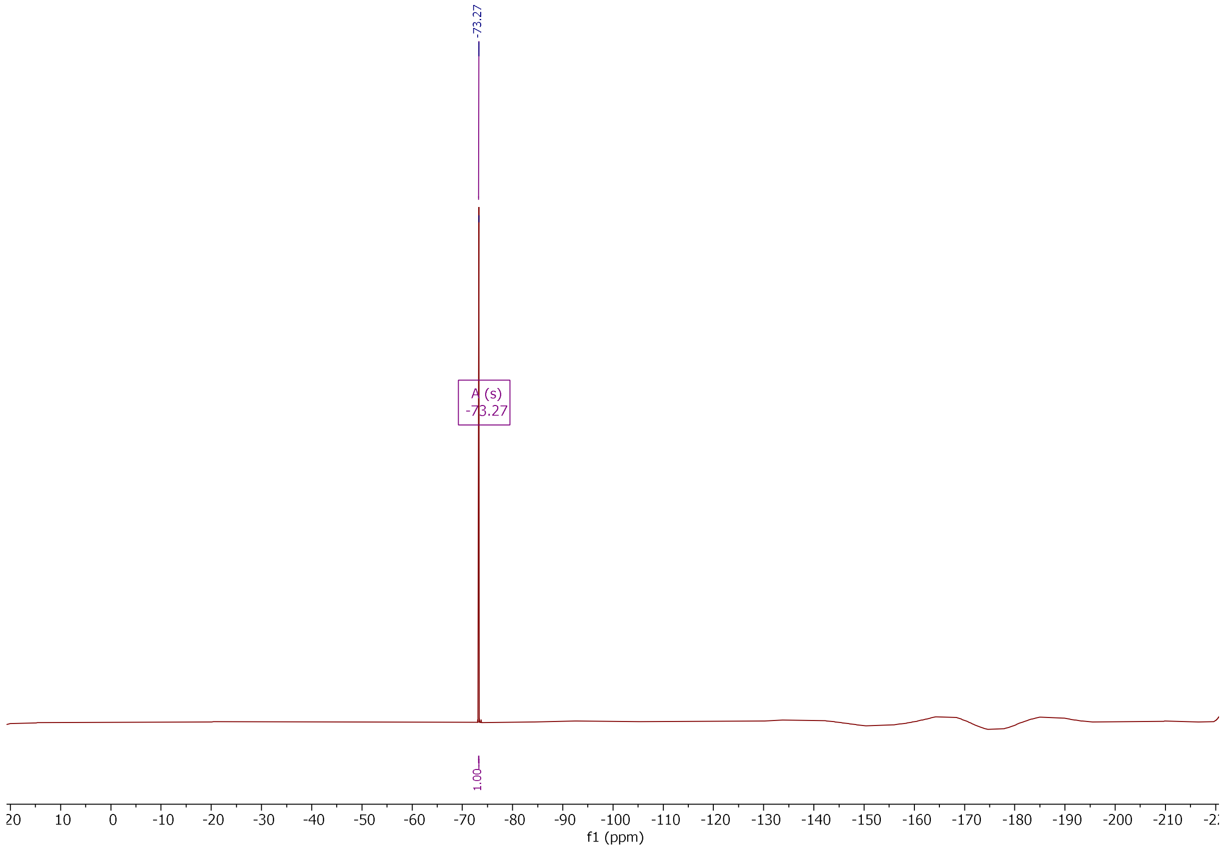

Figure S9 ^{19}F NMR (471 MHz, $CDCl_3$, RT) spectrum of **S5**

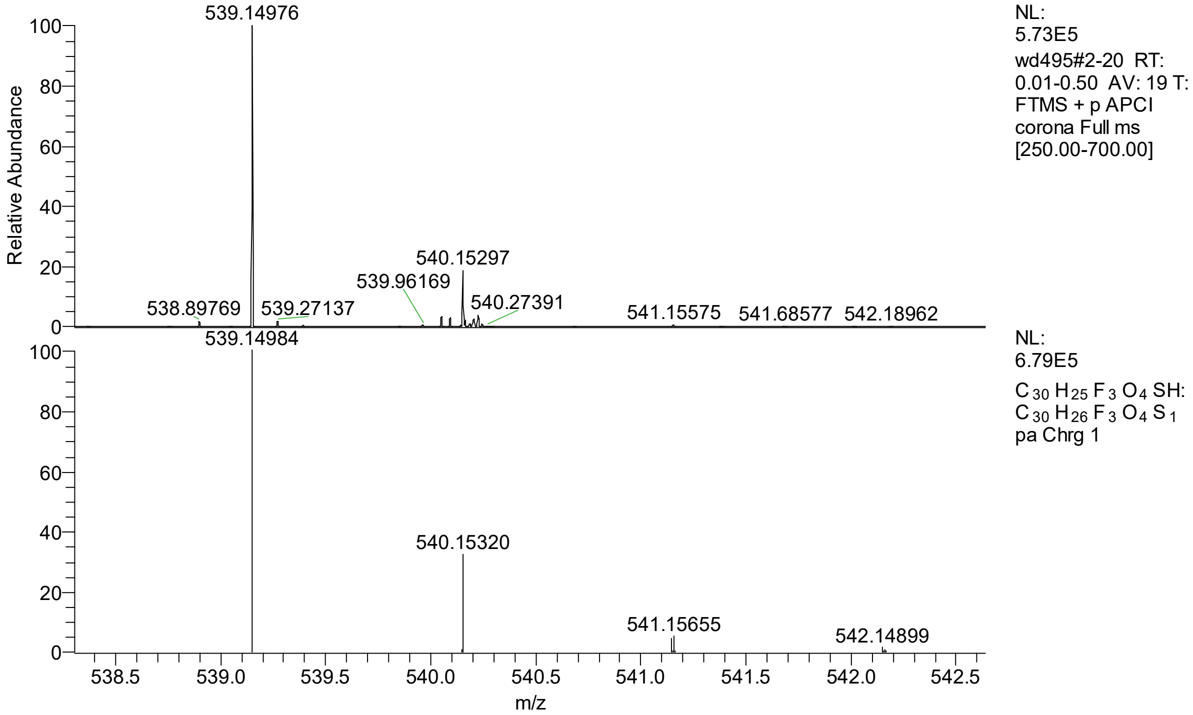

Figure S10 HRMS spectrum of (APCI+) compound **S5** (top: measured, bottom: calculated).

(*Z*)-5'-methoxy-2,2'-dimethyl-2,2',3,3'-tetrahydro-[1,1'-bi(cyclopenta[a]naphthalenylidene)]-5-carbonitrile (**$1GM_{CT}$**)

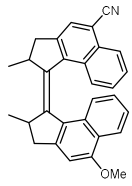

Under nitrogen atmosphere, a Schlenk flask was charged with **S7** (0.75 g, 1.39 mmol, 1.0 equiv.), ^{t}BuXPhos Pd G3 (232 mg, 292 µmol, 0.21 equiv.), ^{t}BuXPhos (219 mg, 515 µmol, 0.37 equiv.) and $Zn(CN)_2$ (327 mg, 2.79 mmol, 2.0 equiv.). Next, a degassed mixture of DMF/H_2O (1% of H_2O *v*/*v*, 10 mL) was added and the resulting solution was heated at 80 °C for 4 h. After cooling to room temperature, the mixture was diluted with 5 volumes of EtOAc, washed copiously with water, brine, dried over $MgSO_4$ and concentrated *in vacuo*. The crude product was purified by flash column chromatography (SiO_2, pentane/EtOAc) and crystallised from EtOH/DCM yielding 0.47 g (1.13 mmol, 81%) of **$1GM_{CT}$** as yellowish crystals.

^{1}H NMR (500 MHz, $CDCl_3$) δ 8.12 – 7.99 (m, 2H), 7.89 (s, 1H), 7.18 – 7.09 (m, 1H), 7.03 – 6.95 (m, 1H), 6.88 (s, 1H), 6.77 (d, J = 8.4 Hz, 1H), 6.53 – 6.45 (m, 1H), 6.45 – 6.34 (m, 2H), 4.07 (s, 3H), 3.70 – 3.49 (m, 4H), 2.77 – 2.61 (m, 2H), 1.30 – 1.16 (m, 6H).

^{13}C NMR (126 MHz, $CDCl_3$) δ 156.8, 146.3, 144.5, 143.5, 142.3, 136.1, 131.7, 130.4, 129.9, 129.5, 128.4, 127.5, 126.8, 126.3, 125.4, 125.3, 125.0, 124.2, 123.9, 122.1, 119.1, 108.3, 102.2, 55.8, 42.4, 42.1, 41.3, 40.1, 21.1, 20.9.

HRMS (ESI+) $[M+H]^+$ calcd for $C_{30}H_{25}NOH^+$ 416.2009, found 416.2005.

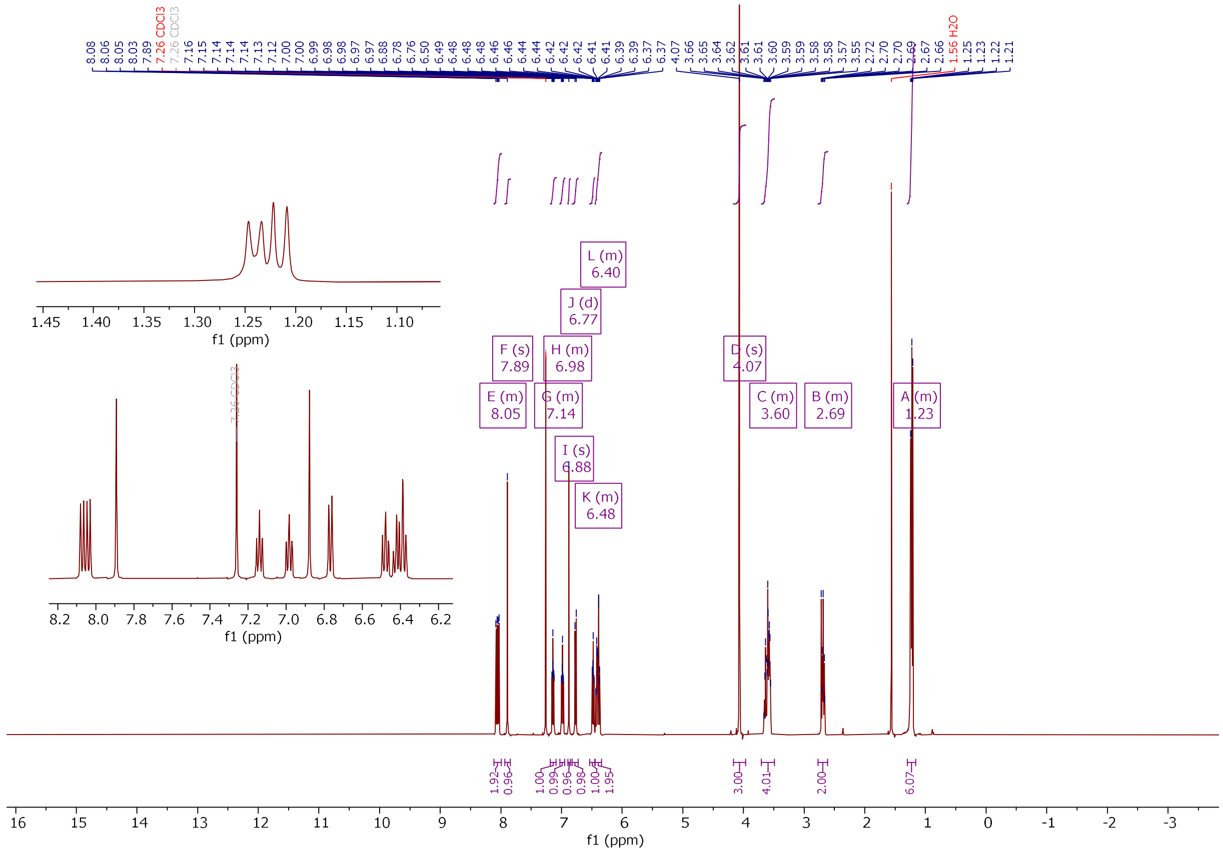

Figure S11 ^{1}H NMR (500 MHz, $CDCl_3$, RT) spectrum of **$1GM_{CT}$**

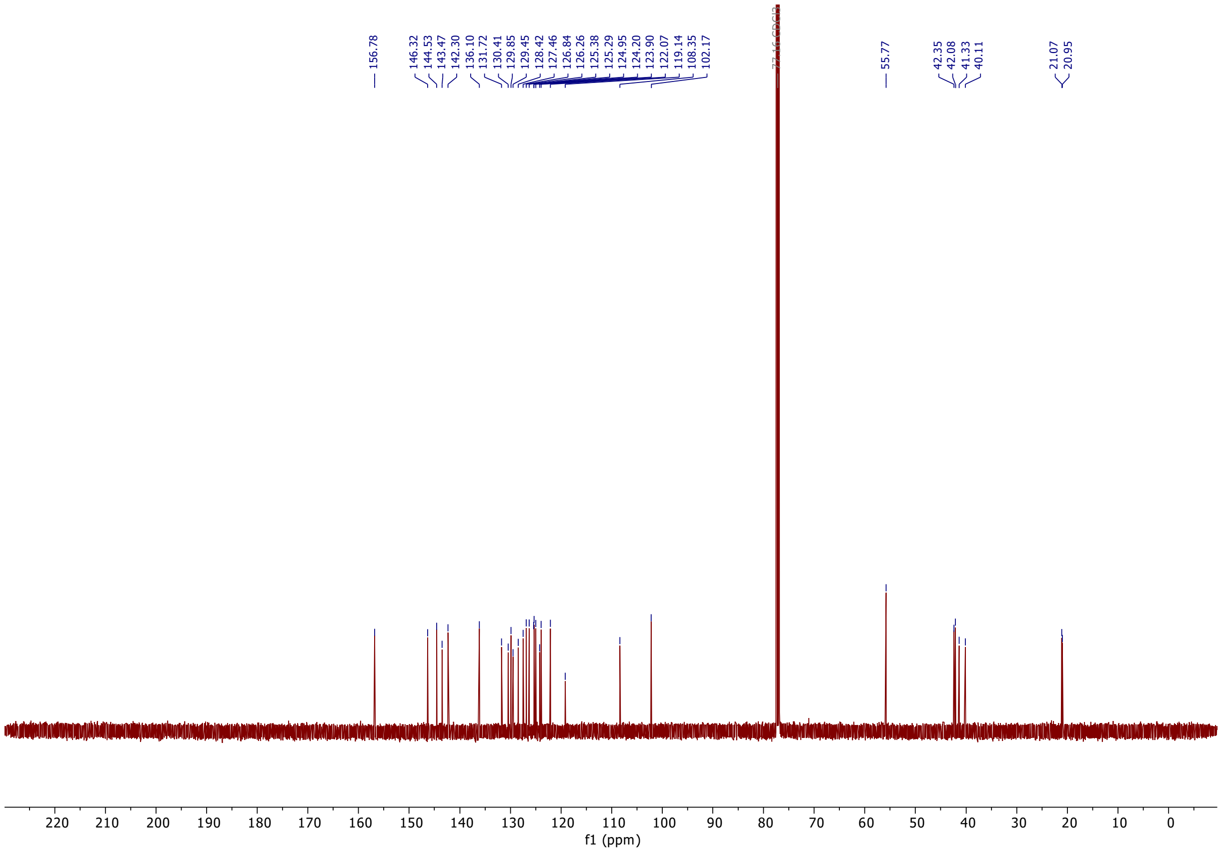

Figure S12 ^{13}C NMR (126 MHz, $CDCl_3$, RT) spectrum of **$1GM_{CT}$**

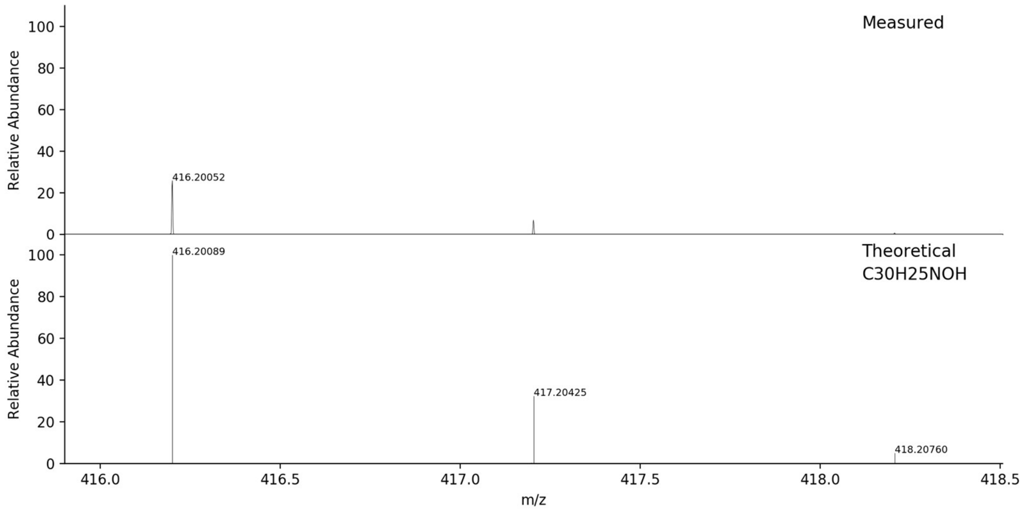

Figure S13 HRMS spectrum of (ESI+) compound **$1GM_{CT}$** (top: measured, bottom: calculated).

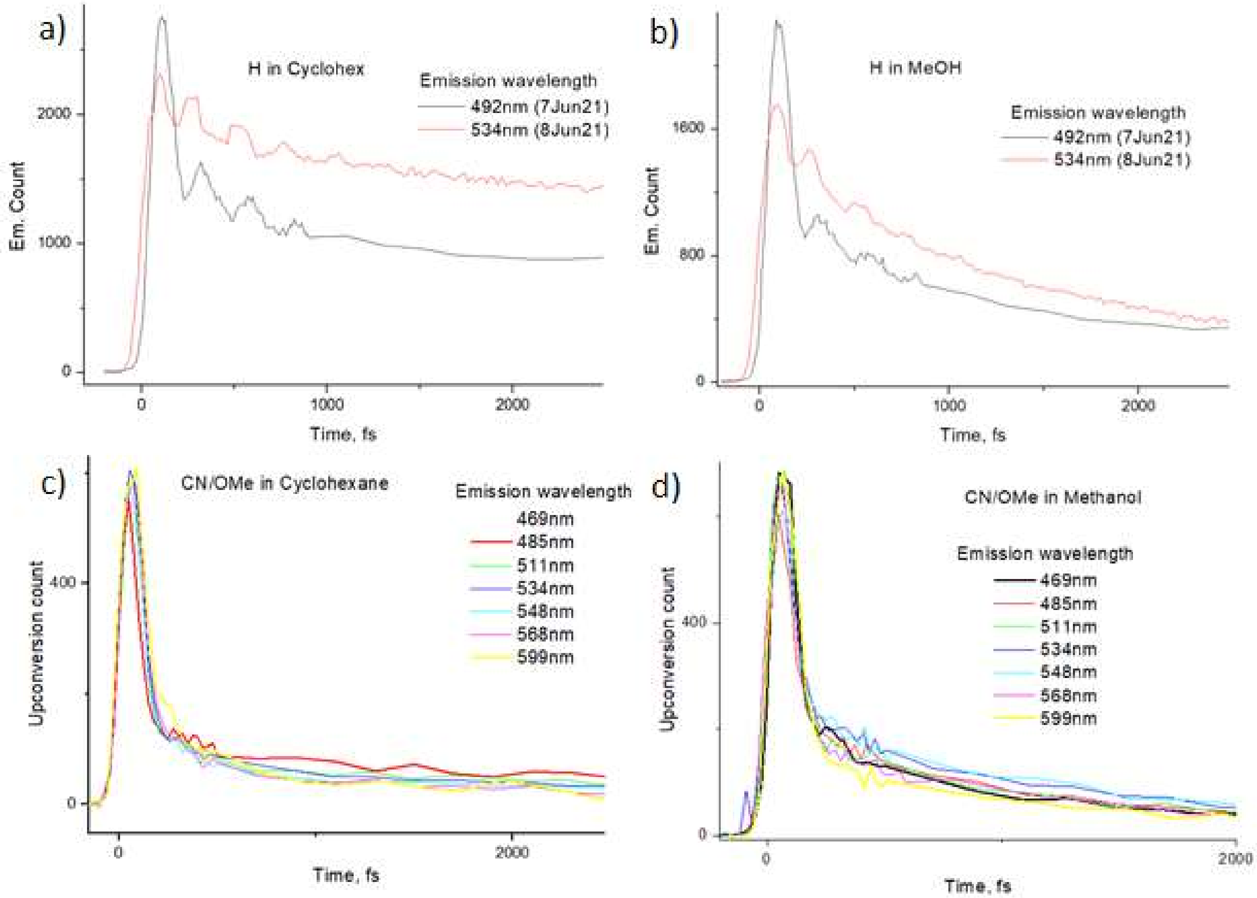

Figure S14. Wavelength dependent fluorescence decay of a) – b) 1GM and c)-d) 1GMCT in cyclohexane and methanol respectively.

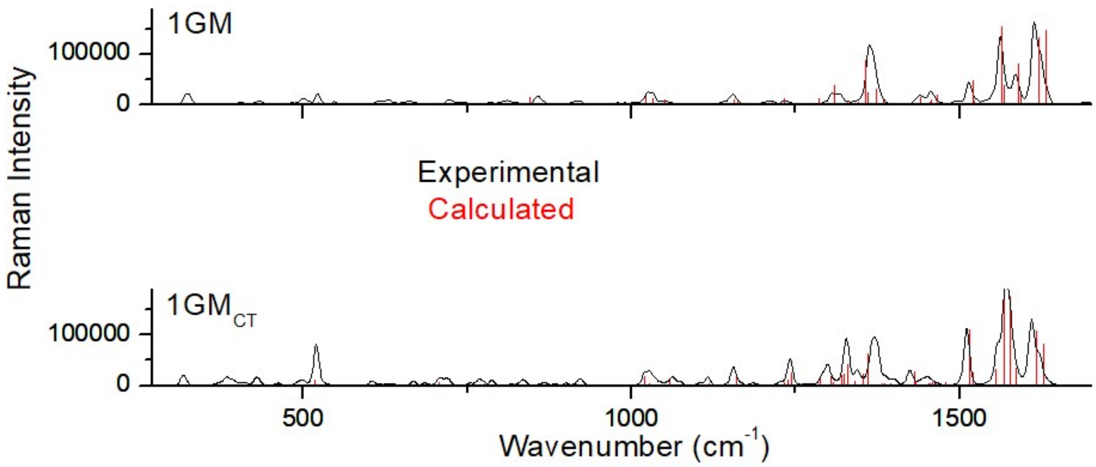

Figure S15. Spontaneous Raman spectra (black) and calculated Raman spectra (red vertical lines) plotted for 1GM (upper) and $1GM_{CT}$ (lower).

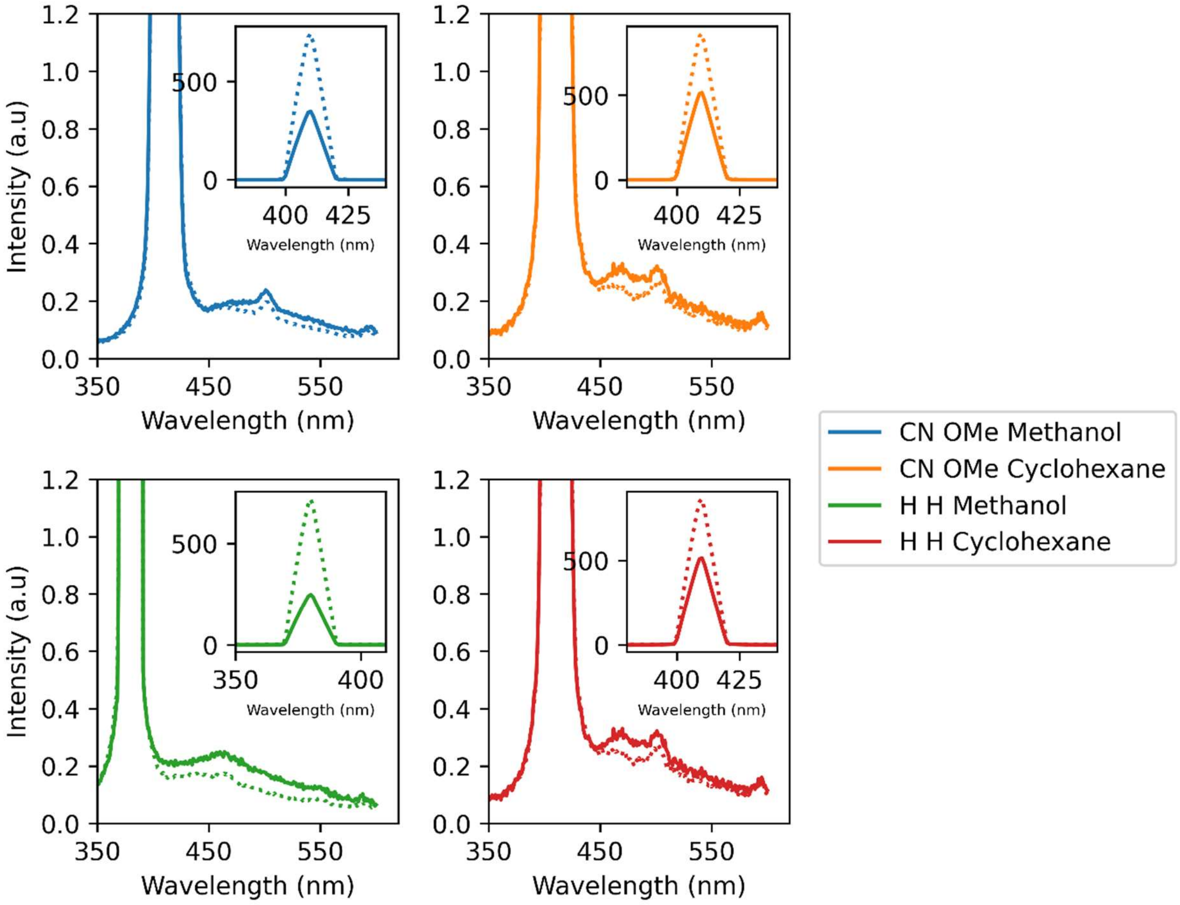

Figure S16. Quantum yields of emission data measured in the integrating sphere. For each sample the reference sample (just solvent) is shown as a dotted line, the excitation band is shown as an inset plot. The Raman bands of solvent can be seen in the spectra near 500 nm. The quantum yield is obtained by integrating the difference between the sample and reference for both the excitation band (inset) and the emission band, and dividing the emission intensity by the amount of excitation intensity missing due to the sample absorption.

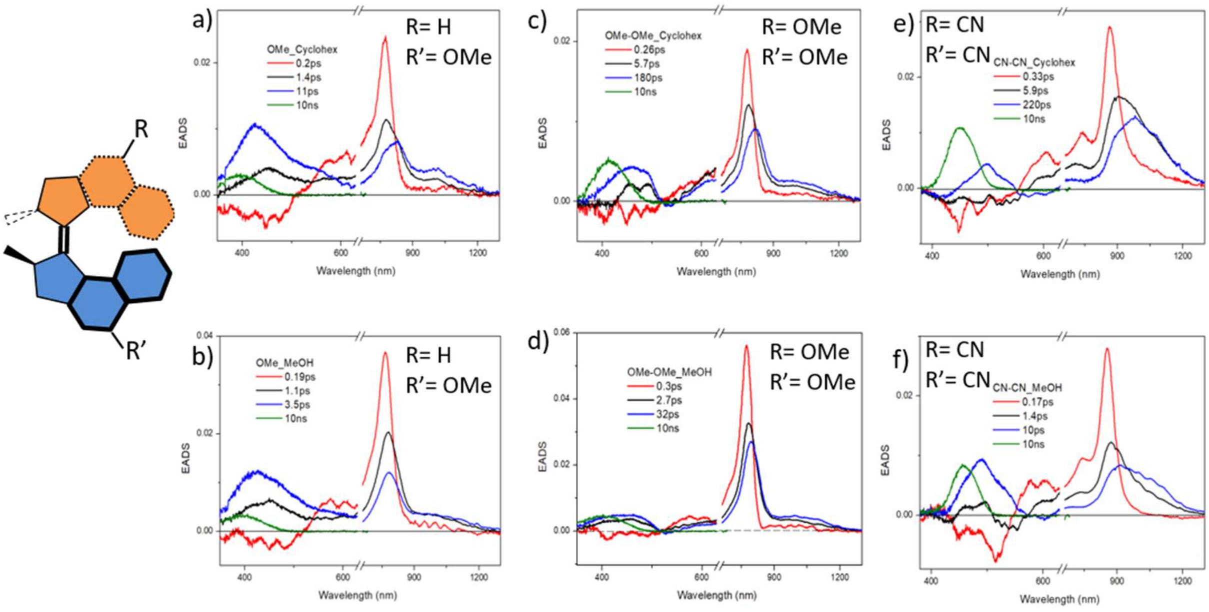

Figure S17 The transient absorption data (Global analyses) are shown for a methoxy, di-methoxy and dicyano derivative of 1GM in cyclohexane a) – c) and methanol d) – f). Detailed analysis (including detail of synthesis) will be presented in a forthcoming paper, but clearly the solvent dependence in yield and the changes in mechanism reported for $1GM_{CT}$ (main paper) is unique among this data set. This is further highlighted in Table S3 below.

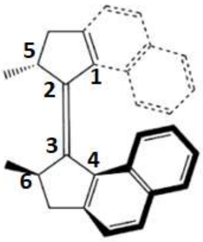

	C2-C3 Bond length	C1C2C3C4 Dihedral angle	C5C2C3C6 Dihedral angle
1GM	1.35342	3.3	17.52
$1GM_{CT}$	1.35536	3.7	17.25

Table S1. Bond length and dihedral angles from DFT optimised structure of motors

1GM methanol	0.3±0.05%
1GM cyclohexane	0.65±0.05%
$1GM_{CT}$ methanol	0.04±0.05%
$1GM_{CT}$ cyclohexane	0.14±0.05%

Table S2 The fluorescence quantum yields expressed as % for 1GM and 1GMCT in methanol and cyclohexane. For details see Figure S16.

**R1**	**R2**	**Relative Yield (TA@ 3ns) MeOH:CHX**
OMe	OMe	1:1
OMe	H	1:1
H	H	1.5:1
CN	CN	0.8:1
CN	OMe	1:6

Table S3. Quantification of the data in Figure S16 where the amplitude of the absorbance at the peak of the FC bright state TA (ca 800 nm at t = 0) to the amplitude of the metastable product at 450 nm was determined. The ratio reported is between that quantity in the two solvents, and is thus representative of the solvent dependent conversion of bright state to product.

Atomic coordinates of Cis-stable 1GM optimized at rb3lyp/tzvp level of calculation:

C	2.58831600	2.37649600	1.26942500
H	3.51089400	2.95669000	1.18908400
H	2.25389500	2.43785100	2.31138800
C	1.97846100	-3.25303200	-1.85450300
C	2.93853600	-2.85527200	-0.96030200
C	2.85894800	-1.59672900	-0.31279000
C	1.72918000	-0.75084000	-0.56799400
C	0.89718400	-2.39736600	-2.14940900
C	3.89936100	-1.14841200	0.53984000
C	1.67634500	0.51133700	0.09975400
C	2.76875000	0.94335200	0.84075900
C	0.67464500	1.59812400	0.05285600
C	-2.76874300	0.94336500	-0.84076100
C	-1.72918500	-0.75083100	0.56799700
C	-2.85895300	-1.59671600	0.31278700
C	-3.89936000	-1.14839700	-0.53984900
C	-2.93854900	-2.85525900	0.96029800
C	-1.97848000	-3.25302300	1.85450500
C	-0.89720300	-2.39735900	2.14941900
H	-3.78845300	-3.49420400	0.74802100
H	-2.05597900	-4.21434400	2.34767400
C	-0.67463700	1.59812800	-0.05285100
C	-1.67634400	0.51134400	-0.09975100
C	-2.58830100	2.37650600	-1.26942800
H	-2.25387400	2.43785800	-2.31138900
H	-3.51087400	2.95670600	-1.18909300
C	3.87220700	0.11093000	1.08608100
C	-3.87220100	0.11094500	-1.08609000
C	-1.47492800	2.88167400	-0.31396600
C	0.77539300	-1.18268200	-1.52146700
C	-0.77540600	-1.18267300	1.52147800
C	1.47494300	2.88166700	0.31396700
C	2.07296400	3.48566200	-0.96586400
C	-2.07294900	3.48567500	0.96586200
H	-0.15645300	-2.70159200	2.87888100
H	0.05594900	-0.53736900	1.76279400
H	-0.05596400	-0.53737800	-1.76277600
H	3.78844000	-3.49421800	-0.74803100
H	2.05595400	-4.21435300	-2.34767300
H	0.15642600	-2.70159900	-2.87886400
H	-4.74178600	-1.80643100	-0.72042800
H	-4.69716600	0.46499400	-1.69380700
H	4.69717600	0.46497600	1.69379500
H	4.74178700	-1.80644800	0.72041400
H	0.85967500	3.63489800	0.80528700
H	2.72644600	2.76859700	-1.46749500
H	2.66228800	4.37619700	-0.73176500

H	1.29249000	3.77487400	-1.67209600
H	-0.85965600	3.63490100	-0.80528700
H	-2.72643600	2.76861700	1.46749300
H	-2.66226600	4.37621400	0.73175900
H	-1.29247500	3.77488300	1.67209600

Atomic coordinates of Cis-stable 1GM$_{CT}$ optimized at rb3lyp/tzvp level of calculation:

C	2.62142000	2.62410300	0.96062000
H	3.53008500	3.19982800	0.76755400
H	2.42036300	2.68276000	2.03632400
C	1.63583100	-2.99635300	-2.07559100
C	2.69305200	-2.60770700	-1.29007500
C	2.68842400	-1.35213400	-0.63950400
C	1.54941100	-0.49527900	-0.76776700
C	0.53871000	-2.13396900	-2.25305800
C	3.82878300	-0.90473000	0.10401000
C	1.56862800	0.77049500	-0.09993600
C	2.73932500	1.19208500	0.50894600
C	0.57447100	1.85780100	-0.02592400
C	-2.94718700	1.22978600	-0.56555600
C	-1.77283600	-0.47892500	0.71698500
C	-2.91327900	-1.33516900	0.57348600
C	-4.04122400	-0.87483700	-0.17715600
C	-2.91400000	-2.59703600	1.21629100
C	-1.86237800	-2.98373200	2.00674100
C	-0.76657200	-2.11941200	2.19922500
H	-3.76985500	-3.24833500	1.09073800
H	-1.88178600	-3.94851900	2.49837000
C	-0.78040400	1.86823700	0.00865400
C	-1.78359400	0.78740000	0.05565000
C	-2.80578900	2.66531200	-1.00130500
H	-2.57913500	2.72948400	-2.07136400
H	-3.71213300	3.24854100	-0.82345700
C	3.86504700	0.36452400	0.64044700
C	-4.06284700	0.40428200	-0.70840900
C	-1.59811000	3.15769200	-0.16095200
C	0.49780200	-0.91866200	-1.61388100
C	-0.72325000	-0.90241500	1.56784000
C	1.40099600	3.13796400	0.15256200
C	1.84198100	3.75404100	-1.18427100
C	-2.05827800	3.75605900	1.17697700
H	0.04594600	-2.41845400	2.84983300
H	0.11905700	-0.24555500	1.72366100
H	-0.34628400	-0.26323900	-1.76687400
H	3.55432100	-3.24965400	-1.17114600
H	1.65372300	-3.95824900	-2.57294400
H	-0.27898600	-2.42895000	-2.89938900
H	-4.94593900	0.75491200	-1.22811700

H	4.74336300	0.73477800	1.15048800
H	0.84932800	3.88569700	0.72179300
H	2.43176000	3.04167800	-1.76510500
H	2.45411800	4.64329700	-1.01316300
H	0.98402400	4.04854300	-1.79118500
H	-1.03371900	3.91142800	-0.70870300
H	-2.66242400	3.04040000	1.73909400
H	-2.66203500	4.65202000	1.01060600
H	-1.20757900	4.03480900	1.80150900
C	-5.17980900	-1.71748400	-0.34871500
N	-6.10316200	-2.39621500	-0.49434400
O	4.84967600	-1.79847500	0.19007400
C	6.03086100	-1.42509800	0.88667800
H	5.81814900	-1.18882100	1.93342100
H	6.68967400	-2.28895700	0.83792800
H	6.51957700	-0.56917900	0.41187900

References

1. Cnossen, A.; Hou, L.; Pollard, M. M.; Wesenhagen, P. V.; Browne, W. R.; Feringa, B. L., Driving Unidirectional Molecular Rotary Motors with Visible Light by Intra- And Intermolecular Energy Transfer from Palladium Porphyrin. *Journal of the American Chemical Society* **2012,** *134* (42), 17613-17619.
2. Pfeifer, L.; Scherübl, M.; Fellert, M.; Danowski, W.; Cheng, J.; Pol, J.; Feringa, B. L., Photoefficient 2nd generation molecular motors responsive to visible light. *Chemical Science* **2019,** *10* (38), 8768-8773.
3. Ji, Y.; DiRocco, D. A.; Hong, C. M.; Wismer, M. K.; Reibarkh, M., Facile Quantum Yield Determination via NMR Actinometry. *Organic Letters* **2018,** *20* (8), 2156-2159.
4. Willett, K. L.; Hites, R. A., Chemical Actinometry: Using o-Nitrobenzaldehyde to Measure Lamp Intensity in Photochemical Experiments. *Journal of Chemical Education* **2000,** *77* (7), 900.
5. Heisler, I. A.; Kondo, M.; Meech, S. R., Reactive Dynamics in Confined Liquids: Ultrafast Torsional Dynamics of Auramine O in Nanoconfined Water in Aerosol OT Reverse Micelles. *J. Phys. Chem. B* **2009,** *113* (6), 1623-1631.
6. Hall, C. R.; Conyard, J.; Heisler, I. A.; Jones, G.; Frost, J.; Browne, W. R.; Feringa, B. L.; Meech, S. R., Ultrafast Dynamics in Light-Driven Molecular Rotary Motors Probed by Femtosecond Stimulated Raman Spectroscopy. *Journal of the American Chemical Society* **2017,** *139* (21), 7408-7414.
7. Snellenburg, J. J.; Laptenok, S. P.; Seger, R.; Mullen, K. M.; van Stokkum, I. H. M., Glotaran: A Java-Based Graphical User Interface for the R Package TIMP. *Journal of Statistical Software* **2012,** *49* (3), 1-22.
8. Frisch, M. J.; Trucks, G. W.; Schlegel, H. B.; Scuseria, G. E.; Robb, M. A.; Cheeseman, J. R.; Scalmani, G.; Barone, V.; Petersson, G. A.; Nakatsuji, H.; Li, X.; Caricato, M.; Marenich, A. V.; Bloino, J.; Janesko, B. G.; Gomperts, R.; Mennucci, B.; Hratchian, H. P.; Ortiz, J. V.; Izmaylov, A. F.;

Sonnenberg, J. L.; Williams; Ding, F.; Lipparini, F.; Egidi, F.; Goings, J.; Peng, B.; Petrone, A.; Henderson, T.; Ranasinghe, D.; Zakrzewski, V. G.; Gao, J.; Rega, N.; Zheng, G.; Liang, W.; Hada, M.; Ehara, M.; Toyota, K.; Fukuda, R.; Hasegawa, J.; Ishida, M.; Nakajima, T.; Honda, Y.; Kitao, O.; Nakai, H.; Vreven, T.; Throssell, K.; Montgomery Jr., J. A.; Peralta, J. E.; Ogliaro, F.; Bearpark, M. J.; Heyd, J. J.; Brothers, E. N.; Kudin, K. N.; Staroverov, V. N.; Keith, T. A.; Kobayashi, R.; Normand, J.; Raghavachari, K.; Rendell, A. P.; Burant, J. C.; Iyengar, S. S.; Tomasi, J.; Cossi, M.; Millam, J. M.; Klene, M.; Adamo, C.; Cammi, R.; Ochterski, J. W.; Martin, R. L.; Morokuma, K.; Farkas, O.; Foresman, J. B.; Fox, D. J. *Gaussian 16 Rev. C.01*, Wallingford, CT, 2016.

9. Neubauer, T. M.; van Leeuwen, T.; Zhao, D.; Lubbe, A. S.; Kistemaker, J. C. M.; Feringa, B. L., Asymmetric Synthesis of First Generation Molecular Motors. *Organic Letters* **2014,** *16* (16), 4220-4223.
